# Stimuli-Responsive Phosphate Hydrogel: A Study on Swelling Behavior, Mechanical Properties, and Application in Expansion Microscopy

**DOI:** 10.1021/acsomega.4c02475

**Published:** 2024-08-28

**Authors:** Yokly Leng, Collin N. Britten, Fatema Tarannum, Kayla Foley, Christopher Billings, Yingtao Liu, Keisha B. Walters

**Affiliations:** † School of Chemical, Materials, and Biological Engineering, University of Oklahoma, Norman, Oklahoma 73019, United States; ‡ Ralph E. Martin Department of Chemical Engineering, University of Arkansas, Fayetteville, Arkansas 72701, United States; § School of Aerospace and Mechanical Engineering, University of Oklahoma, Norman, Oklahoma 73019, United States

## Abstract

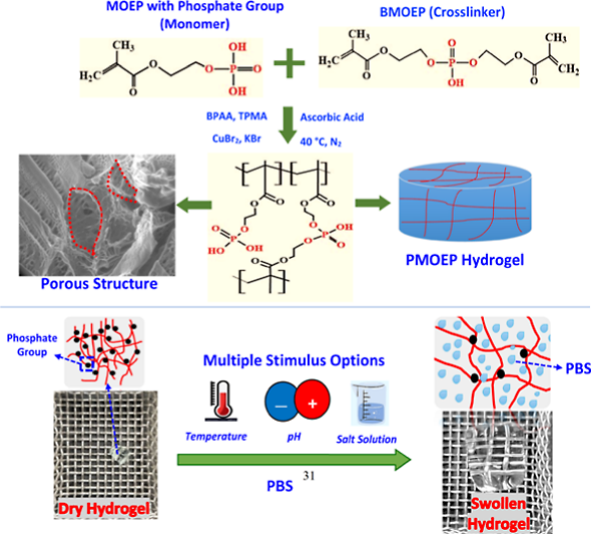

Phosphorus-based stimuli-responsive hydrogels have potential in a wide range of applications due to their ionizable phosphorus groups, biocompatibility, and tunable swelling capacity utilizing hydrogel design parameters and external stimuli. In this study, poly(2-methacryloyloxyethyl phosphate) (PMOEP) hydrogels were synthesized via aqueous activators regenerated by electron transfer atomic transfer radical polymerization using ascorbic acid as the reducing agent. Swelling and deswelling behaviors of PMOEP hydrogels were examined in different salt solutions, pH conditions, and temperatures. The degree of swelling in salt solutions followed CaCl_2_ < MgCl_2_ < KCl < NaCl with a decrease in swelling rate at higher concentrations until reaching a saturation point. In water, the degree of swelling increased significantly around neutral pH and remained constant at basic pH values. The effects of polymerization conditions, including pH, temperature (30, 40, 50 °C), and MOEP concentration (40, 50, 60% v/v MOEP/H_2_O), on the hydrogel swelling behavior in various salt solutions were also investigated. PMOEP hydrogels showed a decrease in the degree of swelling as the pH was increased above the native pH of the monomer solution. Scanning electron microscopy and energy-dispersive spectroscopy were utilized to examine the microstructure and chemical composition of the dried hydrogel after salt solution swelling. Cytotoxicity testing using rat bone marrow stem cells confirmed the biocompatibility of the PMOEP hydrogels. A unique feature of this effort was evaluation of these phosphate hydrogels for use in expansion microscopy where a significant twofold enhancement in cellular expansion capacity was showcased utilizing 4T1 mouse breast cancer cells. This comprehensive study provides valuable insights into the stimuli-responsive behavior and expansion characteristics of phosphate hydrogels, highlighting their potential in diverse biomedical applications.

## 1 Introduction

Stimuli-responsive “smart” hydrogels are three-dimensional (3D) molecular networks composed of covalent bonds and/or noncovalent interactions (e.g., physical entanglements, hydrogen bonds, hydrophilic, supramolecular, electrostatic, and coordination interactions)^1−7^ that exhibit a responsive behavior to environmental stimuli such as temperature,^8^ pH,^9−11^ magnetic fields,^12^ oxidation,^13−15^ and light.^8,16^ Over the past decade, these smart hydrogels have evolved into multiresponsive systems, showcasing their potential for integrated stimulus response and applications in various fields such as tissue engineering, biomedicine, biosensors, and environmental engineering.^17−22^

Phosphorus-bearing hydrogels have attracted significant attention among various stimuli-responsive hydrogels for their wide range of applications in biomedicine and other fields. In biomolecules, such as DNA and RNA, phosphorus groups present in the main chains are fundamental to ensuring the structural integrity and various biological functions, including genetic information storage, transmission, and protein synthesis.^23,24^ For synthetic phosphate-based hydrogels, the anionic phosphate group can provide nucleation and electrostatic binding sites for bone hydroxyapatite calcification and biomaterial adhesion in bone and tissue scaffold applications.^25,26^ Hydrogels containing other phosphorus groups—phosphorylated poly(vinyl alcohol),^27−30^ poly(ethylene glycol) di[ethyl phosphatidyl (ethylene glycol)methacrylate],^31^ phosphoester poly(ethylene glycol),^32−34^ cross-linked poly[2-methacryloyloxyethyl phosphorylcholine]^35−37^—have also been studied for their versatile attributes including biodegradability, biocompatibility, pH and temperature sensitivity, catalytic activity, ion-exchange, and complexing abilities.

In addition to traditional hydrogel parameters (e.g., backbone, cross-linking density, hydrophilicity), the primary contributors to the stimuli response and swelling behavior of phosphorus-bearing hydrogels are the presence of ionizable phosphate (–PO_4_H) or phosphonate (–PO_3_H_2_) functional groups.^38^ Depending on the pH, ion types, and ionic strength, these groups can be (de)ionized, affecting the overall electrostatic interactions and charge distribution within the hydrogel. The change in repulsive forces within the gel leads to changes in the (de)swelling behavior as electrostatic forces either promote the expansion or contraction of the polymer network.^39^ Alterations in temperature can also impact the hydrophilicity of the hydrogel network, leading to phase transitions that impact the hydrogel’s water uptake and swelling properties. The combined pH, ion, and temperature-responsive behavior of phosphate-based hydrogels as well as their biocompatibility have led to several studies focusing on their preparation for biomedical applications.^40−43^ Despite these advancements, very few studies^41,44−46^ have investigated the stimuli response and swelling behavior of hydrogels incorporating the 2-(methacryloyloxy)ethyl phosphate (MOEP) monomer. This highlights a significant gap in understanding the stimuli-responsive behavior, mechanical properties, and applications of MOEP-based hydrogels.

The network structure of the hydrogel also has a significant impact on the swelling and responsive behavior of the hydrogel. Controlled “living” radical polymerizations have shown significant benefits in creating a more homogeneous cross-linked hydrogel network structure, narrower molecular weight distribution, higher degree of chain-end functionality, and well-defined responsive and swelling properties compared to conventional free-radical polymerization. For instance, hydrogels synthesized through both atom transfer radical polymerization (ATRP) and reversible addition-fragmentation chain-transfer (RAFT) techniques have led to greater control over hydrogel properties such as transparency, mechanical modulus, latent end-group functionality, and stimuli-responsive volume change.^4,47−50^

Recently, the authors explored the synthesis of MOEP-based hydrogels through controlled “living” radical polymerization techniques, including activators regenerated by electron transfer atom transfer radical polymerization (ARGET-ATRP) and Cu^0^-mediated ATRP.^51^ These newly developed ATRP techniques focus on the activator regeneration through the introduction of reducing agents rendering ATRP tolerant to a certain degree of oxygen.^52^ In ARGET-ATRP, different reducing agents such as ascorbic acid,^53^ tin(II) 2-ethylhexanoate (Sn(EH)_2_),^54,55^ tertiary amines,^53^ glucose,^56^ and hydrazine^57^ are employed to control catalyst oxidation, ensuring continuous catalyst regeneration through electron transfer at a low concentration of the metal-based catalyst.^2,58^ ARGET-ATRP offers additional benefits, such as minimal side reactions, the capability to be conducted in atmospheric conditions, and compatibility with aqueous phases.^7,59−61^ These features not only facilitate the adjustment of product shape^62^ but also enhance its compatibility with biological systems.^63^

In this study, a phosphate-pendant 2-methacryloyloxyethyl phosphate (MOEP) hydrogel with a bis(2-methacryloyloxyethyl) phosphate (BMOEP) cross-linker was developed using aqueous ARGET-ATRP with an ascorbic acid reducing agent to comprehensively explore its stimuli-responsive swelling characteristics under different factors such as salt solution medium, pH levels, temperature, and synthesis conditions. The study also explores the novel application of the POEMP hydrogel in expansion microscopy (ExM), a recently developed technique where biological samples are fixed in a swellable hydrogel and physically expanded upon swelling.^64−66^ Notably, ExM effectively overcomes the diffraction limit, enhancing the imaging resolution to ∼65 nm with confocal microscopes—4 times better than conventional microscopy alone (∼250 nm).^65,67−69^ POEMP hydrogels offer enhanced biocompatibility and biomaterial adhesion combined with tunable swelling capabilities in different salt, pH, and temperature environments needed for ExM characterization.

Based on the optimized polymerization and swelling conditions in the authors’ previous work, poly(2-methacryloyloxyethyl phosphate) (PMOEP) hydrogels were synthesized with a target degree of polymerization (*D*_P_) of 300. The degree of (de)swelling of PMOEP in different concentrations of NaCl, KCl, MgCl_2_, and CaCl_2_ salt solutions was examined. Additionally, the impact of pH (1.5–10) and temperature (room temperature (RT), 30, and 40 °C) on the swelling behavior was also examined. In order to optimize the hydrogel swelling response and mechanical properties for expansion microscopy, the polymerization conditions were also examined at varying MOEP concentration, pH, and temperature. Scanning electron microscopy (SEM) and energy-dispersive spectroscopy (EDS) were further utilized to confirm the morphology and presence of cations on the surface of dried PMOEP. The mechanical properties and fracture behavior were investigated to further understand the effects of different factors on the degree of swelling. Such in-depth analysis of the MOEP monomer-based PMOEP hydrogel behavior under different stimuli, such as pH, temperature, and exposure to different salt solutions, has not been reported previously. In brief, ARGET-ATRP-mediated PMOEP hydrogel exhibits versatile stimuli-responsive behavior and mechanical properties. Coupled with cytotoxicity studies and a unique expansion microscopy technique, this study provides the background to support these phosphate-based hydrogels in a wide range of applications including controlled drug delivery systems, tissue regeneration, and biomedical engineering.

## 2 Experimental Section

### 2.1 Materials

Phosphoric acid 2-hydroxyethyl methacrylate ester or bis[2-(methacryloyloxy)ethyl] phosphate (BMOEP, C_6_H_10_O_3_·*x*H_3_PO_4_, CAS 52628-03-2; 90%), MOEP (CAS 52628-03-2; 90%, liquid form), potassium bromide (KBr, CAS 7758-02-3, 99%), α-bromophenylacetic acid (BPAA, C_6_H_5_CH(Br)CO_2_H, CAS 4870-65-9; 98%), potassium dihydrogen phosphate, (KH_2_PO_4_, CAS 7778-77-0; 99%), copper(II) bromide (CuBr_2_, CAS 7758-89-6; 98%), potassium chloride (KCl, CAS 7447-40-7), hexane, mixture of isomers (C_6_H_14_, CAS 107-83-5; 98.5%), magnesium chloride (MgCl_2_, CAS 7786-30-3), calcium chloride (CaCl_2_, CAS 10043-52-4), sodium hydroxide (NaOH, CAS 1310-73-2), ascorbic acid (C_6_H_8_O_6_, CAS 50-81-7), proteinase K (cat. no. P4850), gelatin from bovine skin (cat. no. 341635-1MG), Tris base (CAS 77-86-1, cat no. 648311-1 kg), bovine serum albumin (cat. no. A9647-100G), phosphate-buffered saline (PBS, cat. no. P3813-5 × 10PAK), and Corning Costar Transwell cell culture inserts (TC-treated, PET membrane, diameter 6.5 mm, pore size 8.0 μm, sterile, cat. no. CLS3464) were purchased from Sigma-Aldrich. Sodium chloride (NaCl, CAS 7647-14-5, 98%), hydrochloric acid (HCl, CAS 7647-01-0; 36.5–38%), tris(2-pyridylmethyl)amine (TPMA, CAS 16858-01-8, 98%), 18 mm round coverslips (cat. no. 16004-300), and sterile syringe filters (0.2 μm, 25 mm diameter, cat. no. 28143-310) were purchased from VWR. Type 1 ultrapure water was obtained from a Millipore Synergy UV water purification system delivering a pH value of 5.662 and conductivity of 0.055 μS/cm.

Cell freezing medium (cat. no. 12648010), cryovials (cat. no. 12-565-171N), trypsin (cat. no. 25200072), 10% FBS (fetal bovine serum, cat. no. 16000044), 1% penicillin/streptomycin (cat. no. 15140122), α-MEM (minimum essential medium α, cat. no. 12561072), Calcein AM (MW 994.87, lot 2326049, cat. no. L3224), and ethidium homodimer-1 (lot 1976809, cat. no. L3224) were purchased from Thermo Fisher Scientific. Fibroblast growth factor-2 (FGF-2, 2 ng/mL, cat. no. 100-18B) was purchased from PeproTech.

### 2.2 Purification of MOEP

Commercial MOEP contains the cross-linker BMOEP as a production impurity, with BMOEP making up about 25% on a molar basis of the stock MOEP. To reduce this cross-linker concentration, hexane was used to extract BMOEP. As an example, in a 500 mL round-bottom flask, 25 mL of MOEP, 25 mL of type I ultrapure water, and 100 mL of hexane were added and rigorously stirred for an hour using a magnetic stir plate. This mixture was then separated using a separation funnel, as aqueous MOEP/H_2_O will be denser and can be removed from the bottom of the funnel into a clean 500 mL round-bottom flask. This MOEP/H_2_O solution was further purified to extract hexane using reduced pressure rotary evaporation. The volume of the purified solution was measured, transferred to a closed container, and stored in the freezer for later use in polymer synthesis. The concentration of BMOEP in the purified solution is estimated to have been reduced to ∼16% (molar basis) in accordance with the authors’ previous work.^51^ As the BMOEP cross-linker impurity was still present in the purified monomer solution, no other cross-linking agent (e.g., acrylamide) was used in the hydrogel polymerization to avoid introducing other functional groups that could impact the intermolecular interactions within the hydrogel and consequently the swelling behavior. In order to obtain MOEP/H_2_O solutions of various volume ratios (e.g., 60/40, 50/50, 40/60 MEOP/H_2_O), different ratios of MOEP, type I ultrapure water, and hexane were used in the previously described purification procedure.

### 2.3 Synthesis of PMOEP via Aqueous ARGET-ATRP

**Figure 1:**
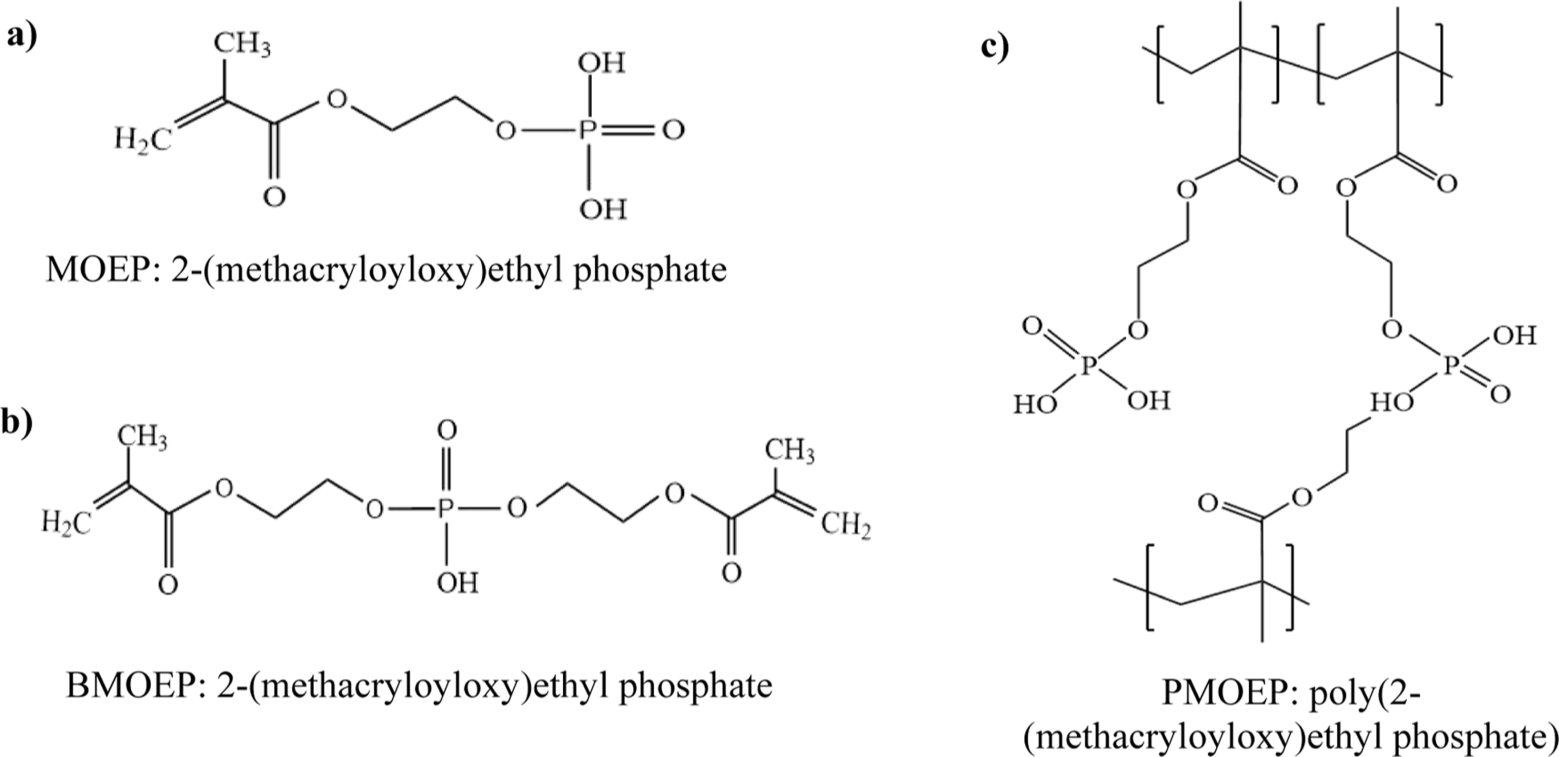
Chemical structures of (a) MOEP, (b) BMOEP, and (c) PMOEP.

**Scheme 1:**
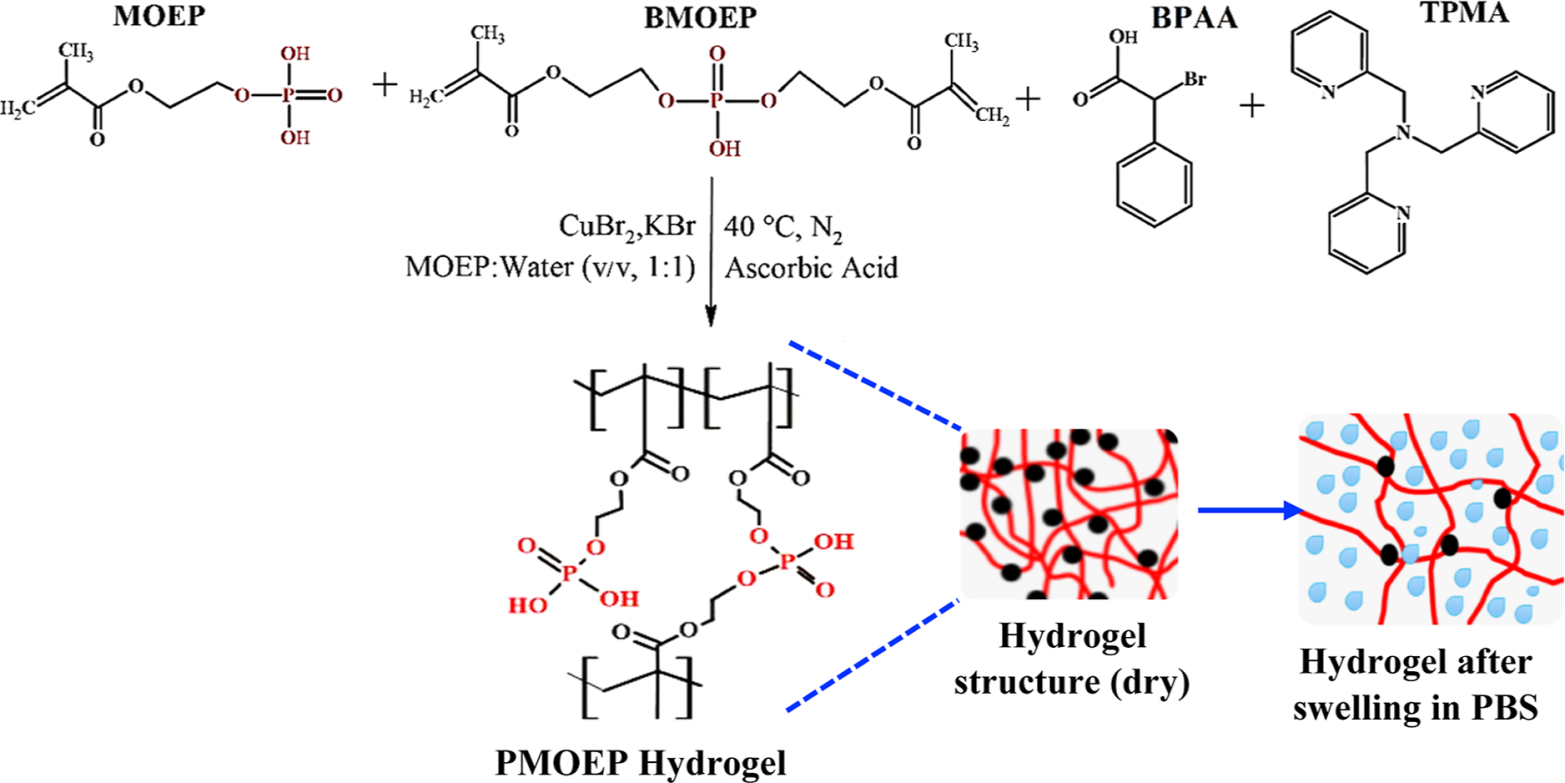
Schematic Illustration of PMOEP Hydrogel Synthesis via ARGET-ATRP Using Ascorbic Acid (AsA) as the Reducing Agent The resulting PMOEP hydrogel structure is composed of poly(methacrylate) backbones (red lines) with pendant and cross-linked phosphate groups (black dots).

Recently, aqueous ARGET-ATRP was used by the authors to polymerize MOEP and cross-link it via its impurity, BMOEP, to form a PMOEP hydrogel.^51^ The chemical structures of MOEP, BMOEP, and PMOEP are shown in Figure 1. In a previous work, the PMOEP hydrogel with the degree of polymerization (*D*_P_) of 300 was shown to be the most stable of those examined with both the highest degree of swelling and the fewest fractures of the gel.^51^ Therefore, in this work, to examine the swelling behavior of the PMOEP hydrogel, the syntheses were designed to achieve *D*_P_ of 300. To obtain *D*_P_ = 300, 9.40 mg of αBPAA, 1.30 mg of TPMA, 1.00 g of CuBr_2_, 59.5 mg of KBr, and 61.5 mg of ascorbic acid were added to a clean and dry 20 mL scintillation vial. Five milliliters of 50:50 by volume of purified MOEP/H_2_O solution was then added to the vial and gently twirled to dissolve the ingredients. The homogenized solution was then transferred to a mold and covered with a glass slide. This mold was then placed in a sealed vessel filled with N_2_ gas. The vessel was then transferred to an oven at 30 °C with continuous N_2_ flow for 8 h to complete the polymerization. The glass cover was carefully removed from the mold, and the cylindrical-shaped hydrogel was removed from the mold for swelling testing. A schematic illustration of the synthesis process is shown in Scheme 1. Hydrogel swelling and equilibration as well as salt solution preparation for induced swelling and swelling are detailed in Supporting Information S1–S3. The procedures for adjusting the pH, temperature of various solutions, and volume compositions under different polymerization conditions are described in Supporting Information S4–S6.

### 2.4 Preparation of Rat Bone Marrow-Mesenchymal Stem Cells with PMOEP Hydrogels for Cytotoxicity Studies

A rat bone marrow-mesenchymal stem cell (rBMSC) was removed from a T-150 flask and transferred to a well plate with α-MEM media and FGF-2. This well plate was then left in the incubator for 24 h to allow the cell to grow. The swollen hydrogels were placed on a glass slide. A 2 mm biopsy punch was used to cut the hydrogel to the desired diameter, and later, the height was adjusted with a sharp blade, appropriate for the Transwell plate. The hydrogel was cured under UV light for at least 15 min. The prepared hydrogel was then added to the Transwell plate and placed on the well with cells at the bottom. α-MEM medium was added to cover the hydrogel. rBMSCs were exposed to the hydrogel for at least 24 h in the incubator.

To kill the cells, 70% methanol was made in a 20 mL vial by adding 700 μL of methanol in 300 μL of PBS. This solution was added to the dead controlled well to kill cells for 30 min in the incubator. All of the wells were then washed with PBS. A live/dead (L/D) assay solution was made with 6 mL of methanol, 3 μL of Calcein AM, and 18 μL of ethidium homodimer-1 in a 15 mL tube. A measure of 0.5 mL of the L/D assay solution was then added to each well and allowed to sit in the incubator for 20 min. Subsequently, the L/D solution was removed from the well plate, and PBS was added. Imaging of live and dead cells in each well was conducted using an EVOS M7000 Imaging System microscope (Thermo Fisher Scientific).

## 3 Characterization

### 3.1 Swelling Kinetics of Hydrogels

The degree of swelling of the hydrogels was determined by immersing the hydrogels in aqueous solutions of the desired pH or other conditions in sealed containers. The hydrogels were removed from the aqueous solution after swelling reached equilibrium and weighed after the removal of excess surface water with a filter paper. The hydrogels were then dried in a vacuum for approximately 24 h, until a consistent weight was obtained.

The degree of swelling is calculated using the swollen weight and dried weight as follows
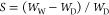
where *S* is the degree of swelling, *W*_W_ is the swollen weight after swelling with PBS and the desired aqueous solution (g), and *W*_D_ is the dried weight (g). Similarly, the rate of swelling is calculated as a percentage using % DS = (*W*_W_ – *W*_D_) /*W*_D_ × 100%. The induced swelling or deswelling rate is calculated using the formula
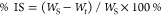
where % IS is the induced swelling or deswelling ratio expressed in percentage, *W*_*t*_ is the swollen weight (g) after 48 h of sample exposure to the salt solution, and *W*_s_ is the initial weight (g) after swelling with PBS and type 1 ultrapure water.

### 3.2 ESEM and EDS Analyses

Environmental SEM (ESEM) (Quattro S FE-ESEM, Thermo Fisher Scientific, Waltham, Massachusetts, USA) was also used to study the morphology of the swollen hydrogel. This ESEM was operated in the secondary electron mode at an accelerating voltage of 20 kV. A small piece of hydrogel was gently punched using a biopsy punch (2 mm in diameter) after swelling with PBS and type 1 ultrapure water. The height of the punched hydrogel was later adjusted with a shape blade to no more than 4 mm and put into the ESEM chamber for a morphological study. In the case of respective salt solutions, the sample was punched by a biopsy punch to 4 mm and cut into three pieces, top facing salt solutions, center of the hydrogel, and bottom exposed to the plate, and then left to dry in a vacuum oven for 24 h to remove water as much as possible to image the surface structure of the dried hydrogel. X-ray EDS was additionally conducted using Quattro S FE-ESEM (Thermo Fisher Scientific, Waltham, Massachusetts, USA) to analyze the elemental distribution, the content of the elements, and material compositions.

### 3.3 Mechanical Properties

The compressive stress–strain measurements were performed by using a Deben in situ Microtest module testing apparatus with a 200 N load cell, a compressive strain rate of 1.5 mm/min, and no preload at RT. The hydrogel samples were 3–3.5 mm in diameter and 2–2.5 mm in thickness. The stress, σ, was calculated by σ = load/π*r*^2^, where *r* is the initial radius of the sample. Strain under compression, ε, was defined as the change in the thickness relative to the thickness of the free-standing specimen.

### 3.4 Expansion Microscopy Imaging of Hydrogels

Zeiss LSM 880 laser scanning confocal microscopes (Zeiss, German) and Keyence BZX microscopes were used to picture the cells before and after expansion. The pre-expansion sample was imaged with a 40×/1.2 NA water immersion objective. The images of the expansion gel were acquired with a 40× air objective. Fluorescent agents 4′,6-diamidino-2-phenylindole (DAPI) and wheat germ agglutinin conjugated with AlexaFluor488 (WGA-AF488) were tagged to the cell membranes in order to provide imaging contrast between the cells and the hydrogel membranes during the laser scanning confocal microscopy analysis.

## 4 Results and Discussion

### 4.1 PMOEP Surface Morphology

**Figure 2:**
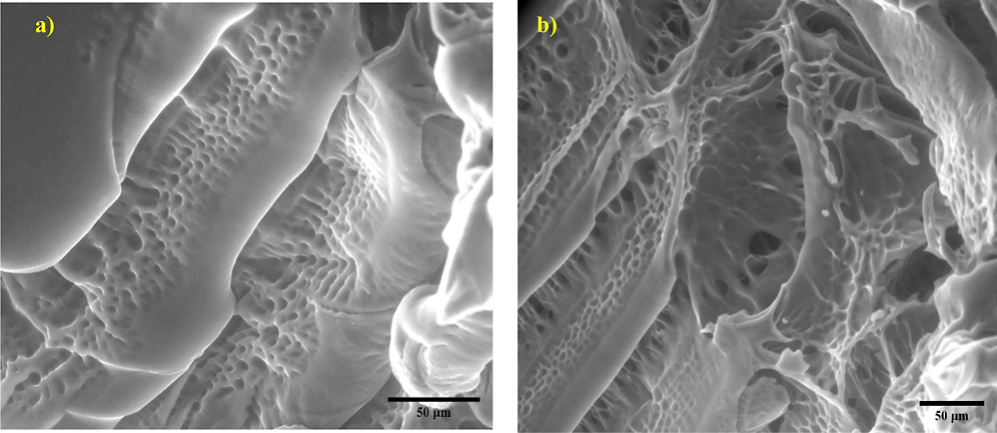
Highly porous network structure of the hydrogel is shown in ESEM images (a,b). The scale bar is 50 μm.

ESEM was used to study the highly porous network structure of the hydrogel, which allows the solvent to diffuse inside. ESEM showed only a small area of the hydrogel as a pressure-limiting aperture was used.^70^ Due to the nature of ESEM, multiple images were captured to show the 3D structure of the hydrogel. Figure 2 presents ESEM images illustrating a complex porous structure of the PMOEP hydrogel. The well-spaced pores within the hydrogel structure demonstrate the overall internal architecture of the hydrogel, reflecting excellent connectivity within the surface of the hydrogel. The ESEM images further emphasize the uniformity and stability of the porous system within PMOEP. The consistency of this well-defined porous structure can play a crucial role in facilitating controlled drug release, making PMOEP particularly promising for applications in drug delivery and other pharmaceutical uses.^71^

### 4.2 Impact of Salt Solutions on Equilibrated Swollen Hydrogels

Similar PMOEP hydrogels have been reported to have the ability to swell more than 6000% of their dried weight when swelling in water and 2500% in PBS.^72^ In general, a balance between the electrochemical potentials of a hydrogel and the chemical potential of a solvent or salt solution is created when an equilibrium is achieved.^73^ However, polyelectrolyte hydrogels are known to have a great response to external stimuli (e.g., solvent solutions).^39^ This means that hydrogels can create a new equilibrium system when exposing equilibrated hydrogels to a new solution, leading to changes in the swelling behavior. For polyelectrolyte hydrogels, the swelling behavior is largely impacted by their ionization degree and the concentration of salts in the aqueous solution in addition to other factors, including pH and temperature.^39^ Depending on the ionization degree, gel deswelling is generally observed upon the exposure of polyelectrolyte hydrogels to increasing salt concentrations due to the electrostatic screening of repulsive interactions between the pendant charged groups of the hydrogel.^74^

**Figure 3:**
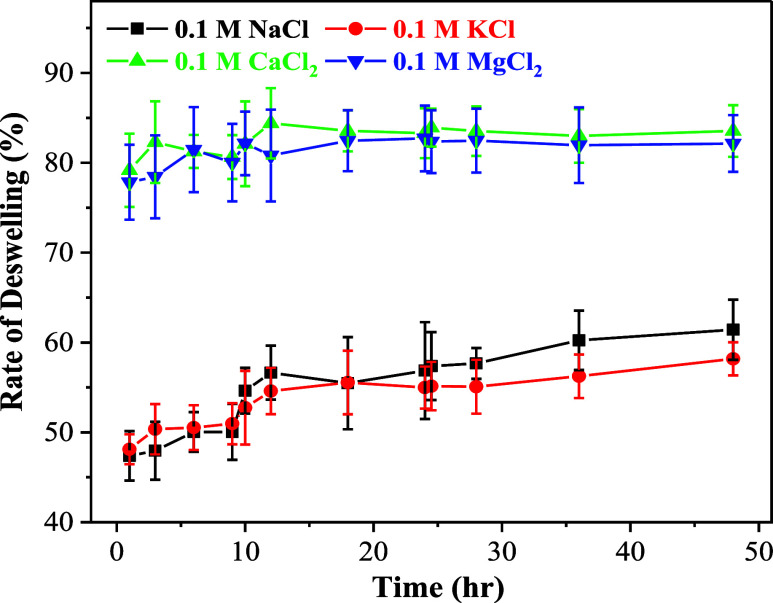
Rate of deswelling of the PMOEP hydrogel (containing 50% MOEP) in 0.1 M salt solutions (NaCl, KCl, MgCl_2_, and CaCl_2_).

The shift in equilibrium of the preswollen PMOEP hydrogel due to exposure to different salt solutions was studied. As the PMOEP hydrogels with a *D*_P_ of 300 were shown to have the highest degree of swelling in water and PBS solution at RT in the authors’ previous work,^51^ the hydrogels were first swelled and equilibrated in PBS and water solution as described in Supporting Information S2. The equilibrated swollen hydrogels were subsequently exposed to different salt solutions (0.1 M NaCl, KCl, MgCl_2_, and CaCl_2_) for 48 h to observe the changes in weight and swelling behavior. Exposing the swollen hydrogel to salt solutions led to a significant decrease in mass for each salt solution examined. Figure 3 shows the rate of deswelling of the PMOEP hydrogel in different salt solutions.

Particularly, 0.1 M CaCl_2_ and MgCl_2_ showed the highest rate of deswelling. This is due to the divalent cations, which could form stronger electrostatic interactions with the phosphate anions of the PMOEP hydrogel compared to monovalent cations such as Na^+^ and K^+^.^75^ This enhanced interaction occurs due to the greater positive charge of divalent cations, leading to stronger electrostatic forces between the cations and the anions in the hydrogel structure. Deswelling occurred mostly within the first hour of the exposure and reached equilibrium after ∼5 h, marked by a plateau in the deswelling rate. A change of salt solutions introduced more cations to the environment around the hydrogel, causing a jump in the deswelling rate at 10 h. A new equilibrium was reached after ∼3 h with these salt solutions. The result showed that 0.1 M CaCl_2_ and MgCl_2_ solutions reduced the mass of the swollen hydrogel by about 80%, while 0.1 M KCl and NaCl solutions could reduce the mass by more than 55 and 60%, respectively. It was also observed that only one out of each of the six samples swelled in CaCl_2_ and KCl, displaying some fractures, while, at minimum, half of the hydrogel samples swelled in NaCl and MgCl_2_ fractured during deswelling.

**Figure 4:**
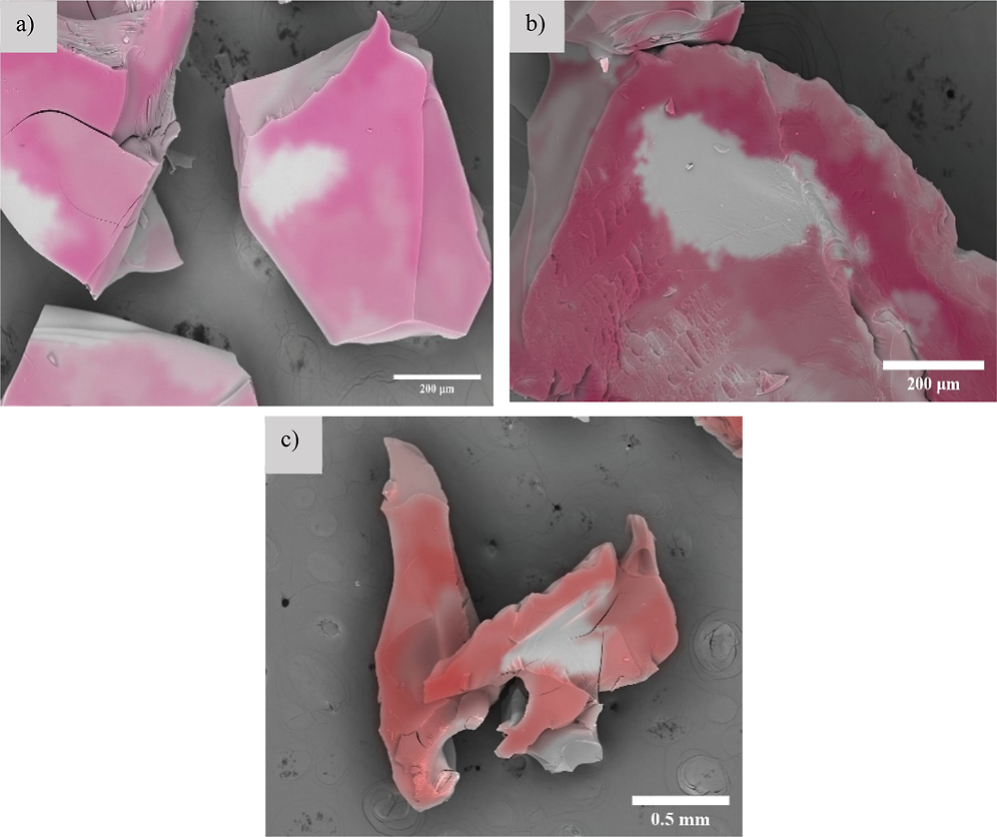
EDS mapping showing the uniform presence of Ca on the (a) surface, (b) center, and (c) bottom of dried PMOEP.

In order to evaluate the transport of cations into the hydrogel after deswelling, EDS mapping was performed and used to determine the salt composition and distribution of the dried hydrogel after deswelling in 0.1 M CaCl_2_ solutions. The result showed a uniform composition of calcium across the hydrogel, as shown in Figure 4. The blank spots appeared due to the angle of the camera on the samples. EDS data indicated that Ca comprised 8.7 ± 0.1 at. % throughout the hydrogel, accounting for approximately 17.93 ± 0.2 wt %. This suggests a consistent presence of Ca within the hydrogel, where, theoretically, calcium interacts with the phosphate pendant group. The combination of the observed deswelling behavior and the uptake of Ca cations in the hydrogel suggests neutralization of the pendant phosphate groups upon exposure of the swollen hydrogels to salt solutions, leading to screened repulsive electrostatic interactions and reduced swell. Detailed EDS spectra and compositions of each component are presented in Supporting Information Table S1.

### 4.3 Effect of Salt Concentrations on the Swelling Behavior

**Figure 5:**
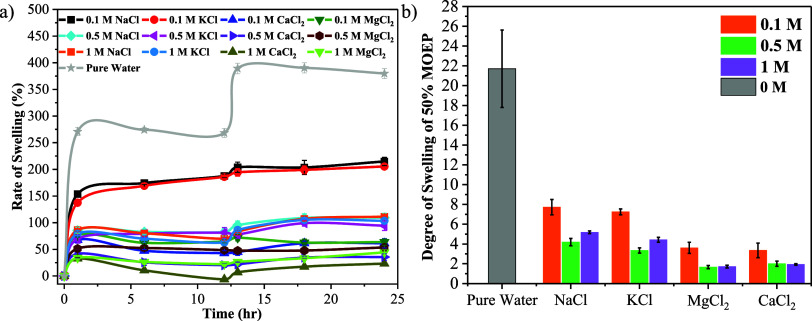
(a) Rate of swelling and (b) degree of swelling of PMOEP after 24 h as a function of salt valency and concentration (0.1–1.0 M).

The impact of different salt solutions and their concentrations on the swelling rate and extent of swelling in PMOEP hydrogels after polymerization was also investigated. In this case, hydrogels produced from 50 v/v % MOEP/water were exposed to different salt types and concentrations, and their rate of swelling profiles and degree of swelling are shown in Figure 5a,b, respectively.

When anionic polyelectrolyte hydrogels are exposed to salt solutions, the salt cations and the pendant anions of the hydrogel interact through electrostatic interactions. This interaction decreases the electrostatic repulsion between anions within the hydrogel network, resulting in a decrease in the osmotic pressure between the hydrogel and the solution. The effect is dependent on the cation type, valency, and concentration in the solution. Monovalent cations result in a greater swelling of the PMOEP hydrogel, overall, compared to divalent cations but less than pure water. This is due to the contributions of charge affecting the swelling behavior of the PMOEP hydrogel. Divalent cations provide a higher positive charge, which increases the strength of electrostatic interactions between the anions of the hydrogel and cations of salt. Consequently, there is a more significant reduction in the repulsion forces between the anions of the hydrogel network, restricting solvent diffusion into the hydrogel. In general, KCl and NaCl (Figures 5a and S1a) solutions yielded similar rates of swelling, while MgCl_2_ and CaCl_2_ solutions had comparable swelling rates (Figures 5a and S1c,d). This indicates that salt solutions with similar valencies (e.g., Na^+^ and K^+^ are monovalent, and Ca^2+^ and Mg^2+^ are divalent) would similarly affect the rate of swelling of PMOEP hydrogels at low concentrations, as shown in Figure 5a.

The PMOEP hydrogel swelling rate (Figure 5a) is also shown as a function of salt type (Figures S1) and concentration (Figure S2) in the Supporting Information document to provide a better understanding to the reader of the connection between the salt concentration, salt type, and the corresponding swelling rate. Figure S2a,b shows the swelling behavior of PMOEP in KCl and NaCl salt solutions at different concentrations. Lower salt concentrations correspond to higher swelling rates. However, as the concentration of the salt increases, the swelling behavior of the hydrogel is not significantly impacted. Notably, at 0.5 and 1 M concentrations (Figure S2a,b), the swelling behaviors are closely aligned. Figure S2c,d shows the swelling behavior of PMOEP in MgCl_2_ and CaCl_2_ solutions, with the rate of swelling decreasing with an increased salt concentration. Noticeably, both Mg^2+^ and Ca^2+^ show decreases in the swelling rate after an hour. A possible explanation is that divalent cations diffuse into the swollen hydrogel, interact with the anions of the hydrogel, and lead to electrostatic screening of the pendant anionic charges, as was observed for the deswelling behavior of the pre-equilibrated hydrogels in Section 4.3. Moreover, divalent cations can chelate both the pendant phosphate group and the ester (C=OO) group of the methacrylate backbone within the hydrogel network, causing a further restriction in the polymer chain motion and in swelling. In particular, at high Ca^2+^ concentrations, the hydrogel collapses, indicating strong interactions between Ca^2+^ and the hydrogel. These interactions may be strong enough to push the aqueous MOEP solution from the hydrogel, causing a further decrease in volume, even to values below the initial hydrogel volume. This effect is also observed, albeit to a lesser extent, for MgCl_2_, NaCl, and KCl at higher salt concentrations (above 0.5 M for MgCl_2_ and 1 M for NaCl and KCl). Increased cation diffusion into the hydrogel and condensation of divalent metal cations and high concentrations of monovalent cations can eventually lead to charge reversal of the polymer chains after binding.^39,76^ This, in turn, leads to an increase in charge repulsive interactions between the positively charged metal-bound polymer chains and subsequent reswelling. The larger initial deswelling response of the hydrogel in CaCl_2_ solutions compared to MgCl_2_ may be attributed to the differences in the hydration shell and the metal cation coordination with the phosphate group (e.g., bidentate vs monodentate).^77−80^ After an initial rapid swelling (*t* < 1 h), the sample’s water uptake was relatively stable until ∼12.5 h. The inflection observed at this point in the swelling rate occurred just after the immersion solution was replaced with fresh salt solution which reset the concentration of the solution back to the original concentration.

Figure 5b shows that NaCl induces the highest degree of overall swelling in PMOEP at 50% v/v of MOEP/H_2_O, followed by KCl, MgCl_2_, and CaCl_2_ salt, respectively. This suggests that monovalent cations have weaker interactions with the pendant hydrogel anions, resulting in a lower intermolecular force. This allows more water to diffuse into the hydrogel. In contrast, divalent cations such as Ca^2+^ and Mg^2+^ provide a higher positive charge and, therefore, stronger electrostatic interactions. This limits the ability of water to diffuse into the hydrogel as the interaction also affects the pore size and space within the hydrogel network. Typically, an increase in salt concentration would allow more cations to interact with the hydrogel network, resulting in reduced repulsive interactions between the pendant anions and lower water diffusion into the hydrogel. Similarly, a decrease in the degree of swelling is observed for all salt solutions when the concentration is increased from 0.1 to 0.5 M (Figure 5b). However, increasing the salt concentration from 0.5 to 1.0 M for CaCl_2_ and MgCl_2_ solutions did not affect the degree of swelling significantly. This can be attributed to the fact that the saturation of each cation occurs uniformly across the hydrogel network as a whole, rather than being limited to only on the surface.

### 4.4 Effect of pH of Immersed Medium on the Swelling Behavior

Most hydrogels are pH-responsive^81^ where the pH of immersion medium has a direct influence on the swelling behavior of the network. An effort to understand the swelling behavior of PMOEP in solutions with different pH values at RT was conducted. pH values of 1.5, 4, 6.5, 7.4, and 10 were chosen based on the p*K*_a_ values of the phosphate pendant group, which have been reported at p*K*_a1_ = 4.5 and p*K*_a2_ = 7.7.^82,83^

**Figure 6:**
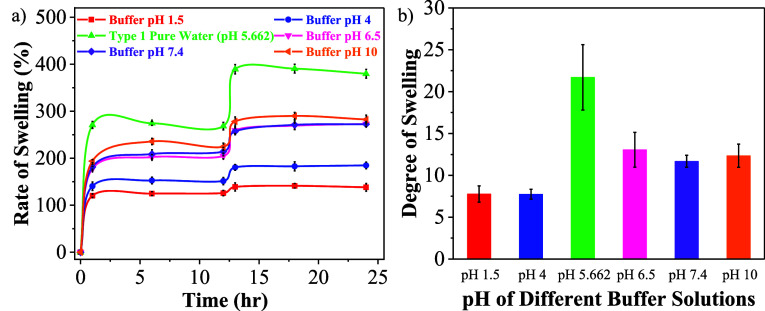
(a) Rate of swelling and (b) degree of swelling of PMOEP (containing 50% MOEP) as a function of solution pH.

Detailed information regarding the pH adjustments is described in Supporting Information S4. Figure 6a illustrates that water (pH 5.662) shows the highest rate of swelling due to the hydrogen bonding between water molecules and the PMOEP network, creating more space for water to swell, thus leading to a higher swelling rate. The increase in pH results in an increase in the swelling rate; however, the buffer solutions still had a lower swelling rate compared to pure water, likely due to charge-shielding ion species present in the buffer solutions. At low pH, protonation of the phosphate groups of PMEOP results in fewer negatively charged groups in the hydrogel, leading to reduced repulsive interactions and a lower swelling rate. In contrast, basic solutions have an increased concentration of OH^–^ species and increased deprotonation of the PMEOP group which help to increase the repulsion forces between anionic groups on the polymer chains. As a result, basic solutions showed a higher rate of swelling compared to acidic solutions. Similar behavior has been observed for anionic polyelectrolyte hydrogels such as those containing carboxylic acid groups.^84^

Type 1 ultrapure water shows the highest degree of swelling, while the pH 6.5 buffer shows the highest degree of swelling among other pH-buffered solutions (Figure 6b). However, the degree of swelling became nearly constant when pH was increased from 7.4 to 10 or 1.5 to 4. This shows that the degree of swelling of MOEP is relatively unaffected at extreme pH levels, either acidic or basic. However, PMOEP shows a substantial change in the degree of swelling, particularly around neutral pH.

### 4.5 Effects of Temperature, pH, and MOEP/H_2_O Volume Compositions on the Swelling Behavior

**Figure 7:**
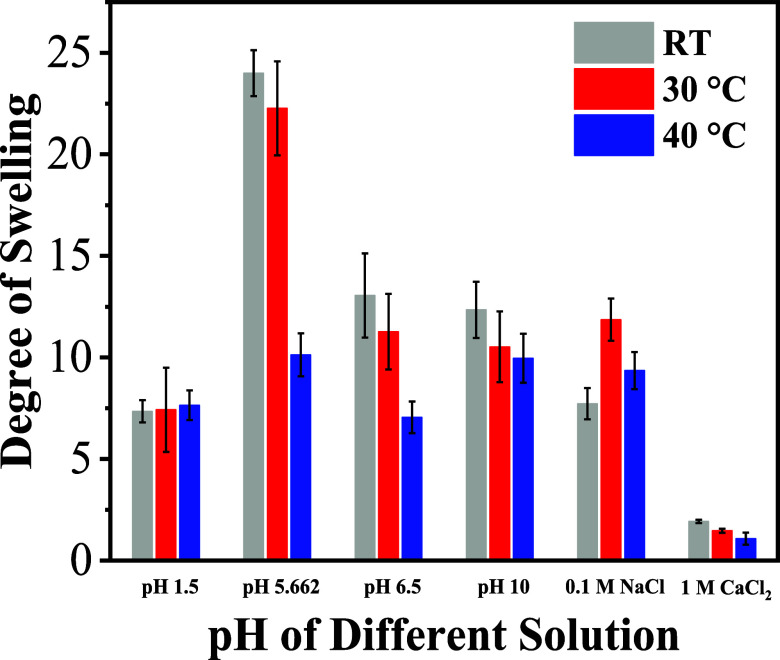
Degree of swelling of PMOEP containing 50% MOEP in PBS solutions at different pH and salt solutions for different swelling temperatures.

The swelling behavior of PMOEP hydrogels as a function of different solution concentrations and pH at different temperatures has also been investigated as shown in Figure S4 for the rate of swelling. Additionally, Figure 7 represents the degree of swelling of PMOEP hydrogels containing 50% v/v MOEP/H_2_O after swelling in different solutions, including those at buffer pH 1.5, 6.5, 10, type 1 ultrapure water, 0.1 M NaCl, and 1.0 M NaCl and at temperatures of RT, 30, and 40 °C. The temperature adjustment process for selected salt solutions and different pH values is described in Supporting Information S5. The swelling behavior of a polymer with increasing temperature can be categorized into one of the three categories: (1) swelling increases with temperature (e.g., poly(dihydroxypropyl methacrylate), (2) swelling decreases with increasing temperature (e.g., poly(hydroxypropyl acrylate), and (3) the combination of (1) and (2) (e.g., poly(2-hydroxyethyl methyl acrylate) (PHEMA).^85^

In our case, PMOEP falls into category (2). In general, an increase in temperature results in a decrease in swelling rate, although this change was not as significant for increases in temperature between RT and 30 °C. Noticeably, type 1 water and buffer pH 6.5 show otherwise (Figure S4b,c). The swelling rate decreases significantly for buffer solution pH 1.5 at a higher temperature (Figure S4d); however, there is no noticeable change in the degree of swelling with the increasing temperature. The lower swelling rate could result from a lower mass of the hydrogel in general, resulting in reduced water absorption. Similarly, type 1 ultrapure water and buffer pH 6.5 show a large decrease in swelling rate at 30 °C as well (Figure S4c). However, the overall trend in the degree of swelling remains consistent, exhibiting a decrease as the temperature increases (Figure 7). While the critical solution behavior of the hydrogels as a function of temperature was not formally studied in this work, the decreasing degree of swelling in aqueous solutions with increasing temperature in the pH range from ca. 5.7 to 10.0 suggests that the PMOEP hydrogel exhibits increasing hydrophobicity or a denser network with increasing temperature. However, as no opacity was observed during the studies, it is likely that the LCST of the hydrogel is above 40 °C or that the hydrogel is exhibiting a broad thermal transition as has been observed for several cross-linked hydrogel systems.^86−89^ In the case of swelling in 0.1 M NaCl PMOEP (Figures S4e and 7), the degree of swelling was the highest at 30 °C before dropping at 40 °C. At low pH (∼1.5), the temperature did not significantly impact the degree of swelling, as a similar swelling behavior was observed for each temperature.

**Figure 8:**
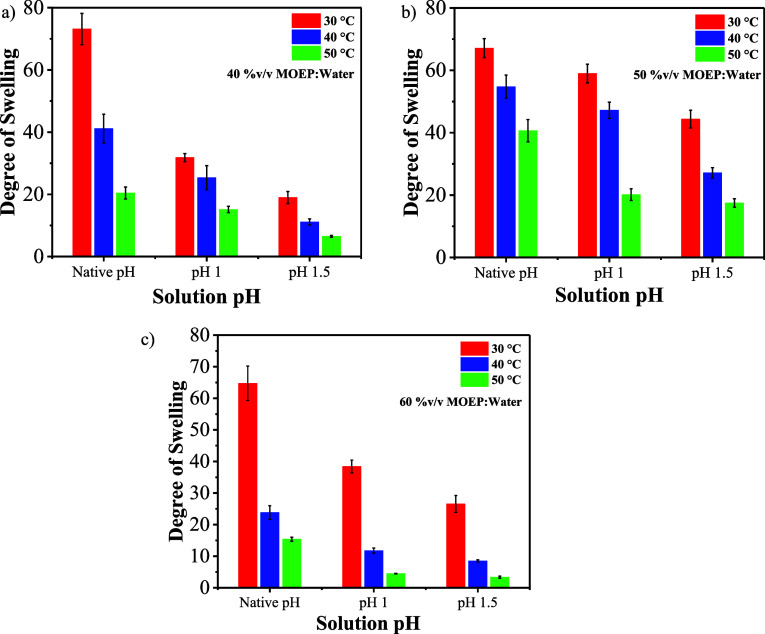
Degree of swelling at RT for hydrogels synthesized with (a) 40% MOEP, (b) 50% MOEP, and (c) 60% MOEP by volume, corresponding to native solution pH values of 1.04, 0.7, and 0.4, respectively. Both polymerization solution pH and temperature inversely impact the degree of swelling observed in the PMOEP hydrogels.

Figure 8 extends the analysis of the swelling behavior of different polymerization conditions with distinct MOEP contents, pH values, and at temperatures of 30, 40, and 50 °C. The overall trend shows that an increase in the polymerization condition pH decreases the degree of swelling mainly when pH is changed from the native pH of the composition to pH 1 (Figure 8). The pH adjustment process of MOEP solutions at different volume compositions is described in detail in Supporting Information S6. At a low MOEP content (40% v/v) polymerized at 30 °C, the degree of swelling significantly drops when the pH slightly decreases from its native pH (1.04) to pH 1 (Figure 8a). The degree of swelling drops from ∼73 to ∼32, more than half of the degree of swelling of MOEP polymerized at its native pH. This result can be explained from the interaction between Na^+^ cations and the anion groups of PMOEP, causing an increased pressure within the PMOEP network and therefore a reduction in the ability of water to diffuse into the network (i.e., reduced osmotic pressure). Moreover, the presence of Na^+^ can result in ionic cross-linking which then reduces the ability of water to swell into PMOEP. However, Figure 8b shows that at 30 °C, 50/50 (v/v %) of MOEP/H_2_O at native pH (0.7) results in the degree of swelling of ∼67, which dropped to ∼44 when the pH is increased to 1.5. At a higher temperature (50 °C), the degree of swelling significantly decreases, dropping from ∼40 at native pH (0.7) to ∼17 at pH 1.5 (Figure 8b).

Overall, the degree of swelling shows an inverse trend with the increase in the polymerization temperature and pH value (Figure 8). While the degree of swelling drops very significantly for 40/60% v/v of MOPE/H_2_O and 60/40% v/v of MOEP/H_2_O, the decline is not as substantial for 50/50 (v/v %) MOEP/H_2_O gel where the degree of swelling dropped from ∼67 at 30 °C (pH 0.7) to ∼55 at 40 °C (pH 1) and ∼44 at 50 °C (pH 1.5). The increase in the polymerization temperature indicates an increase in the reaction rate, resulting in a less organized hydrogel structure. Moreover, the temperature increase could also affect the hydrogel’s properties. It was also observed that at a high MOEP concentration (60/40% v/v of MOEP/H_2_O) and 50 °C, the resulting hydrogel gave a light brown color, which can be attributed to the oxidation of copper at that temperature. Similar color was also observed for 50/50% v/v of MOEP/H_2_O solution (Supporting Information, Figure S5).

### 4.6 Fracture Behavior and Mechanical Properties of PMOEP Hydrogel

**Figure 9:**
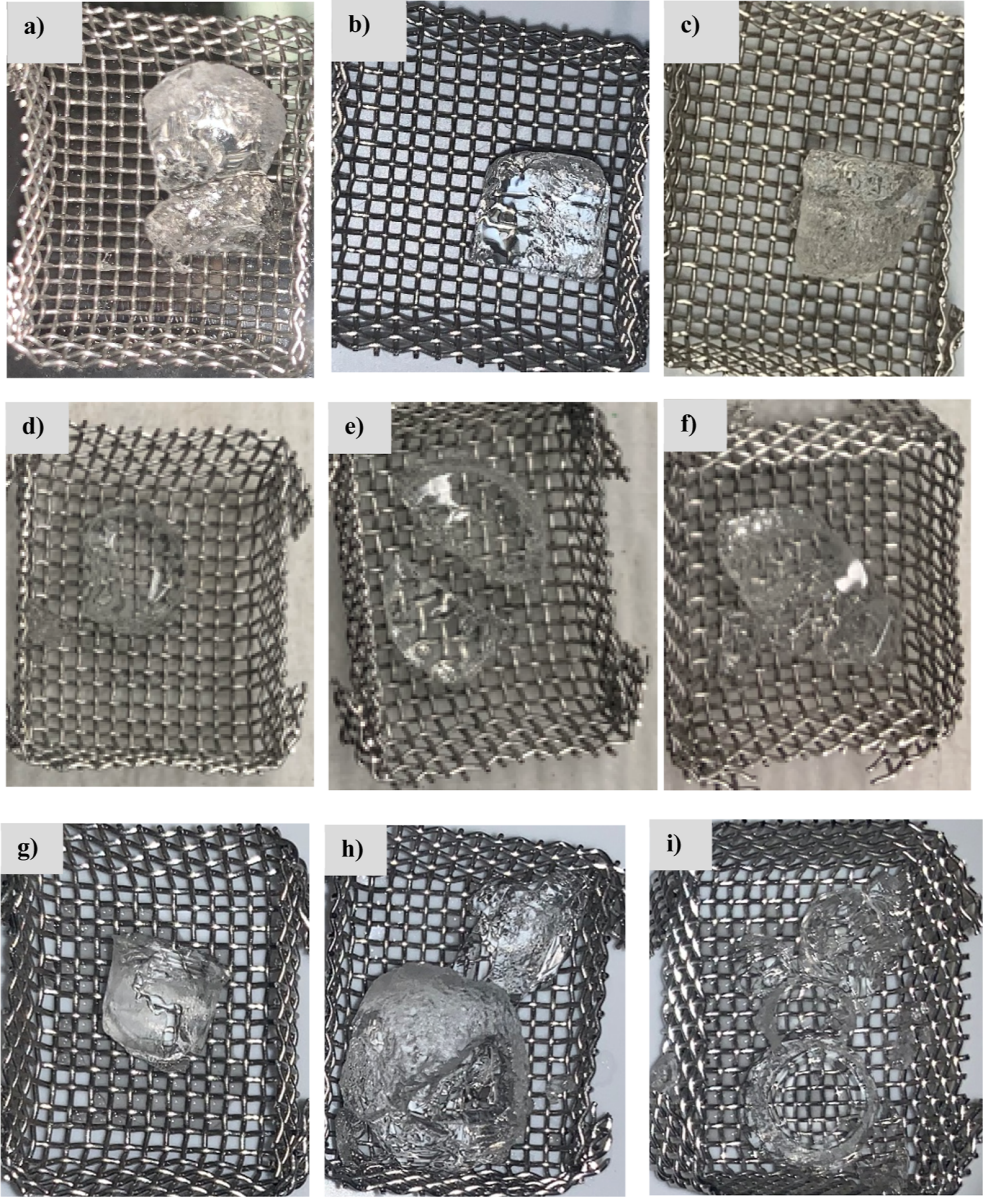
PMOEP hydrogels containing 50% MOEP after swelling: (a) 40 °C water (some fracturing), (b) PBS pH 10, and (c) 0.1 NaCl solution. Swollen PMOEP hydrogel synthesized at 50 °C containing 60% MOEP: (d) native pH (0.4), (e) pH 1.0, and (f) pH 1.5. Swollen 50% MOEP hydrogels: (g) native pH (0.7), (h) pH 1.0, and (i) pH 1.5—all showing fracturing.

The fracturing behavior of PMOEP hydrogels during swelling was reported for lower degree of polymerization samples in the authors’ previous work.^51^ It was observed that fracturing may occur when the polymerization was performed at different temperatures, pH values, and MOEP concentrations. Noticeably, PMOEP containing 50/50% v/v of MOEP/H_2_O cracked when swelling in water at 40 °C (Figure 9a). However, no fractures in the hydrogels were observed for PBS at pH 10 or 0.1 M NaCl solutions (Figure 9b,c). As shown in Figure 11d–f, the swollen hydrogel synthesized at 50 °C displayed various degrees of fracture, ranging from minimal to significant fracture for 60/40% v/v of the MOEP/H_2_O composition. PMOEP synthesized at pH 1.5 and 50 °C was nearly completely destroyed after swelling in water, while at lower pH the shape remained intact with only some minor fractures. PMOEP synthesized at 50 °C with 50/50% (v/v) of MOEP/H_2_O fractured (Figure 9g–i); however, the extent of fracturing decreased with increased pH.

**Figure 10:**
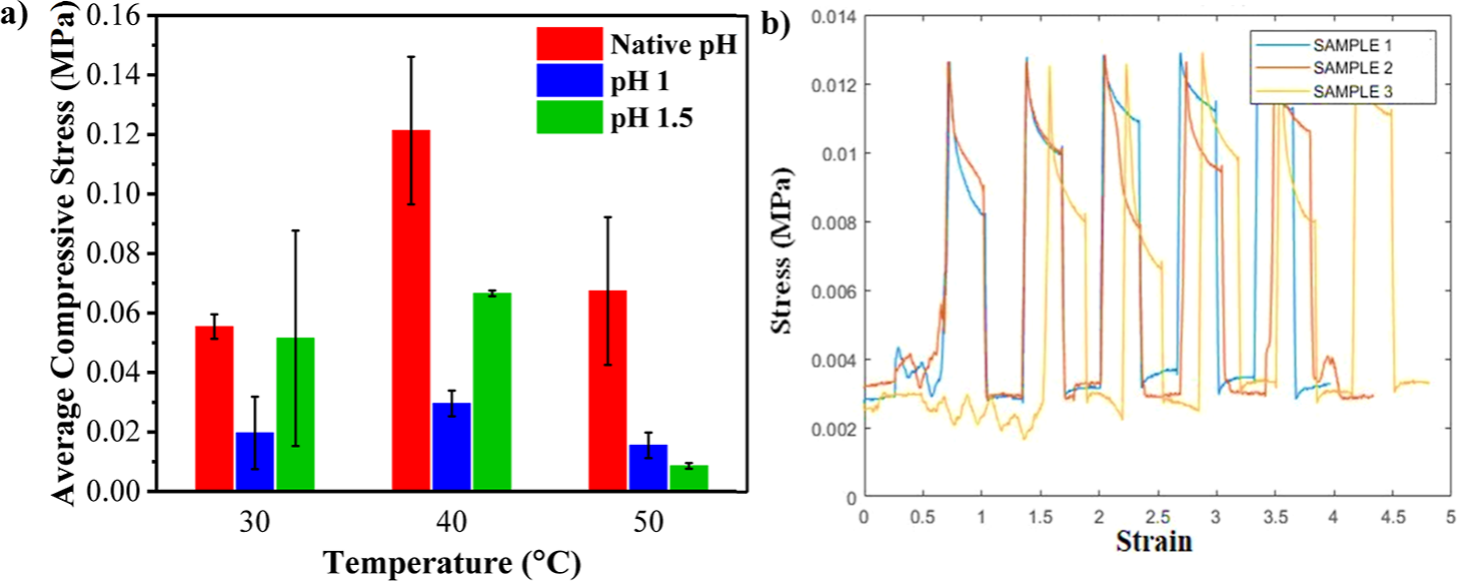
(a) Average compressive stress of PMOEP samples polymerized with 50% MOEP at 30, 40, or 50 °C and at native (0.7) or elevated (1.0 and 1.5) pH conditions. (b) Stress–strain cycles for PMOEP hydrogels polymerized at 40 °C and 0.7 pH.

PMOEP hydrogels made from 50/50% v/v of MOEP/H_2_O compositions were chosen for additional mechanical property testing due to their regular and repeatable formation of cylinder samples. Increasing the polymerization pH, up to a certain level, decreased the overall compressive stress in the resultant hydrogel samples. As shown in Figure 10a, the average compressive stress was significantly reduced upon increasing the native pH (0.7) to 1 and further increased at pH 1.5 for samples polymerized at 30 and 40 °C. For 50 °C, there is a rapid decrease in compressive stress upon increasing the pH to 1, followed by a slight reduction with a further increase to pH 1.5. For all three pH values tested, the compressive stress was at the maximum at a polymerization temperature of 40 °C. For instance, when polymerized at 30 °C and native pH (0.7), the compressive stress is 0.0554 MPa, more than doubles to 0.1213 MPa at 40 °C, and decreases to 0.0674 MPa at 50 °C (Figure 10a). The highest compressive stress is achieved at 40 °C at its native pH (0.7). Cyclic stress–strain testing of the PMOEP hydrogels was conducted for different pH values and temperatures. These hydrogels were shown to withstand repeated applied force, as long as the force was low enough to prevent the fracture of the hydrogel. Figure 10b illustrates this result, showing PMOEP hydrogel samples (pH 0.7 and 40 °C) enduring compressive stress of 0.012 MPa applied for five cycles without any fractures or irreversible deformation.

### 4.7 Cytotoxicity of PMOEP Hydrogels

**Figure 11:**
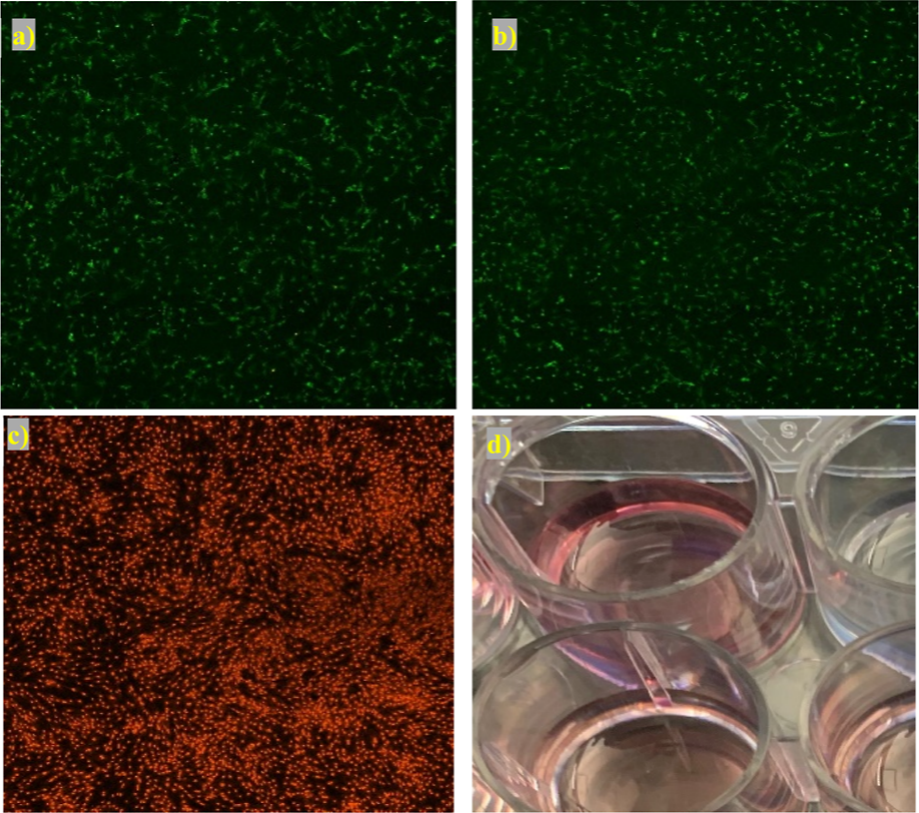
Images showing live (green) and dead (red) rBMSCs upon exposure to PMOEP hydrogel (polymerized at 50 °C and pH 1.5) at (a) 0 and (b) 24 h. (c) Control sample showing dead rBMSCs killed by 70% methanol in PBS. (d) Cell growth media color change during the cytotoxicity test; top well was the negative control and bottom well was exposed to hydrogel.

The cytotoxicity of PMOEP was examined with rBMSCs via leaching tests. The rBMSC cells were exposed to PMOEP hydrogels and allowed to grow under normal conditions for 24 h in an incubator. Figures 11a–c and S9 display images showing live and dead rBMSCs after exposure to hydrogels polymerized at various temperatures and pH values. Comparing Figure 11a,b, very few cells were killed during the 24 h test period, with the cells continuing to grow as usual. This suggests that PMOEP allows for a prolonged cellular contact, at least 24 h, without significant cytotoxic effects. The observed low cytotoxicity, coupled with sustained cell growth, emphasizes the potential of PMOEP for a wide range of biomedical applications, where a prolonged contact with cells is required, such as drug release systems and contact lenses. As a control, at the end of the cytotoxicity testing, the rBMSCs were killed by exposure to methanol; this sample showing red/dead cells is shown in Figure 11c. During the exposure of the cells to the hydrogels, it was noted that the media pH decreased, as indicated by the change of the media to a lighter shade of pink (Figure 11d). This may have been due to the use of type 1 ultrapure water (pH ca. 5.6) to swell the hydrogel prior to testing.

### 4.8 Expansion Microscopy Imaging of PMOEP

**Scheme 2:**
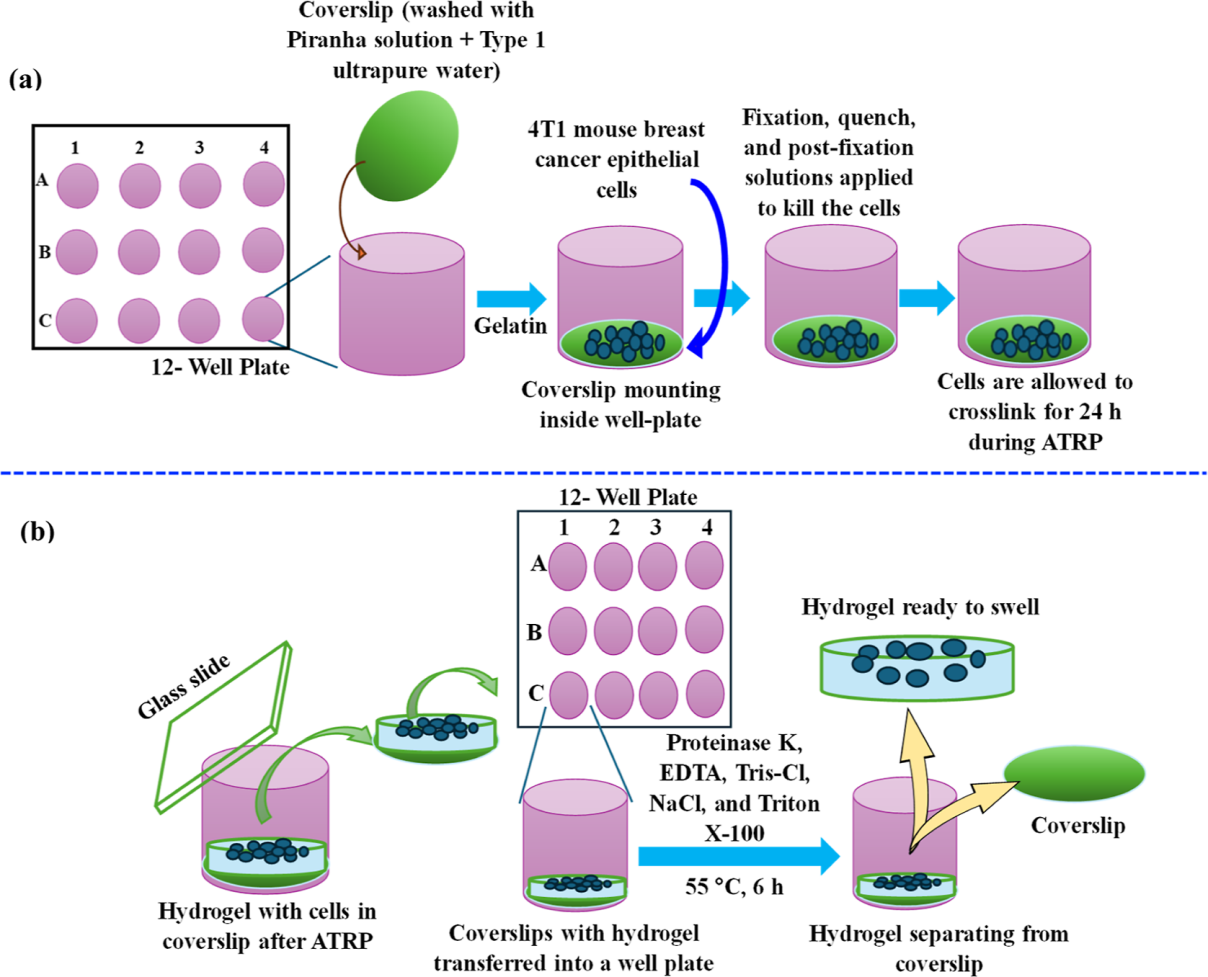
Schematic Illustration of (a) Coverslip Preparation for Cell Seeding in ATRP and (b) Cell Digestion of Hydrogels

**Figure 12:**
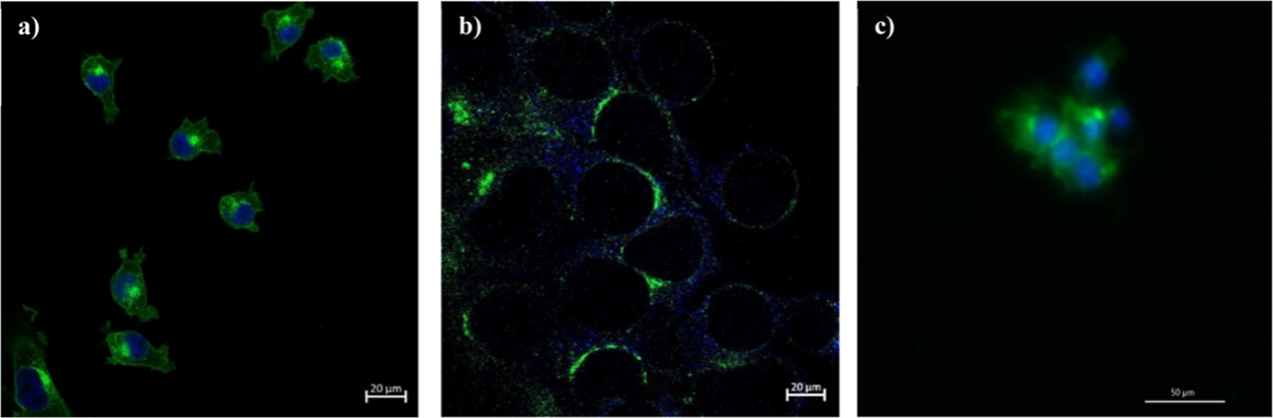
Images of 4T1 cells: (a) pre-expansion, (b) post-expansion (Zeiss LSM 880 laser scanning confocal microscope using 40× water objective, NA = 1.2), and (c) post-expansion with digestion (Keyence BZX microscope using 40× air objective).

The PMEOP hydrogel was examined as a substrate for expansion microscopy (ExM). ExM was performed using the PMEOP hydrogel synthesized via aqueous ARGET-ATRP from 50% v/v MOEP/H_2_O with a target *D*_P_ of 300. Preparation of the coverslips for cell seeding as part of the PMOEP polymerization and cell digestion procedures is shown in Scheme 2 and described in Supporting Information S7 and S8. Unsaturated carbons in the lipid bilayer are capable of cross-linking with some proteins, allowing the PMOEP hydrogel network to cross-link with the cell membrane. Since cells can integrate into the PMOEP network structure, as the hydrogel swells, the cells also expand. The goal of ExM is to expand the cells, especially those with limited size, and take advantage of isotropic expansion for improved imaging. Figure 12a shows the diameters of 4T1 cells before expansion. The exact increase in diameter of the 4T1 cells could not be accurately measured due to the excessive background signals and autofluorescence in the postexpansion cell images. There are three possible reasons for the reduction in image brightness observed: (i) the polymerization could have damaged the fluorophores; (ii) the dye was lost during the digestion process; and (iii) the dye was diluted during the expansion.^90^ Overlapping of DAPI and WGA AF488 signals can prevent distinguishing the nuclei and membrane from other cell components. Regardless, based on the image in Figure 12b, it was estimated that PMOEP swelling expanded the 4T1 cells by twofold. Light scattering can occur due to a mismatch of the refractive indices (RI) between water in the hydrogel network (RI ∼ 1.33) and the coverslip (RI ∼ 1.58), which results in spherical aberrations.^91^ Another limitation with ExM is difficulty with microscope imaging, especially if a large expansion moves the surface to be imaged far away from the coverslip. In an attempt to improve the image, the hydrogel with integrated cells was digested in a protein enzyme (proteinase K) solution for 6 h on a 55 °C hot plate before expansion. This resulted in a significant improvement in the image quality but at the expense of a reduced expansion, as shown in Figure 12c. The digestion step likely damaged the bond between the cell and the hydrogel network, resulting in little cellular expansion when the hydrogel was swollen. Overall, the combined ability of the PMEOP hydrogels to withstand repeated force applications, its tunable swellability in different solution environments, low cytotoxicity, and the twofold cell expansion capabilities indicates favorable potential for the use of the PMEOP hydrogels as novel expansion agents for expansion microscopy and other biomedical applications.

## 5 Conclusions

In this study, poly[2-(methacryloyloxy)ethyl phosphate] (PMOEP) hydrogels were synthesized via aqueous ARGET-ATRP using BMOEP moieties as the cross-linker from a commercially available phosphate monomer, MOEP. The swelling behavior of the hydrogel showed significant responsiveness to various stimuli: type I ultrapure water; different salt species and concentration solutions; pH; and temperature. The rate and degree of swelling were the highest in water, while swelling in salt solutions was substantially lower, following the trend Na^+^ > K^+^ > Mg^2+^ > Ca^2+^, due to stronger interactions between divalent cations (Ca^2+^ and Mg^2+^) than monovalent cations (Na^+^ and K^+^) and the hydrogel network. SEM and SEM–EDS analyses confirmed the presence of cations in the dried hydrogel. Polymerization conditions, including temperature (30, 40, and 50 °C), MOEP concentration (40, 50, and 60% by volume), and pH, were varied to study the impact on the resultant PMOEP hydrogel’s swelling properties. Higher reaction temperature, monomer concentration, and pH resulted in a decrease in the degree of swelling. PMOEP hydrogels showed the highest degree of swelling when 40% MOEP solution was polymerized at 30 °C at its native pH (1.04). Fracturing behavior was observed across all samples polymerized at 50 °C; this lower stability may be attributed to the denser hydrogel network as the polymerization kinetics is increased at higher temperatures. It was found that PMOEP polymerized from 50% MOEP at 40 °C at its native pH (0.7) could maintain its physical form and withstand the highest strain without fracturing. Cyclic mechanical testing also showed no irreversible deformation to this sample when force was applied for five cycles. After the PMOEP hydrogels were found to not exhibit cytotoxicity, they were synthesized in the presence of 4T1 mouse breast cancer cells on a coverslip to examine their potential in expansion microscopy imaging. The PMOEP swelling allowed for an approximately twofold growth in the cell size, revealing a preliminary but unique feature. The stimuli-sensitive phosphate-based PMOEP hydrogels demonstrated excellent controlled swelling and mechanical properties, showcasing their potential in precise drug delivery, environmental sensing, corrosion protection, and smart textile applications.

## Supporting Information Available

Swelling and equilibration of hydrogels, induced swelling of PMOEP, preparation of salt solutions, pH adjustments of PBS and MOEP solutions with different volume compositions, temperature adjustment for selected salt solutions and pH, preparation of coverslips for and use for cell seeding in PMOEP polymerization, cell digestion of hydrogels, DSC analysis, rate of swelling of PMOEP in different concentration solutions, images showing the coloring of PMOEP after polymerization at 50 °C with different pH values, images of live and dead rBMSCs after exposing to hydrogels synthesized at different polymerization conditions, average EDS data for different salt solutions at different concentrations, and SEM images showing potassium salt precipitation on dried PMOEP hydrogels after swelling (PDF)
