# Supplementary material for: Stimuli-Responsive Phosphate Hydrogel: A Study on Swelling Behavior, Mechanical Properties, and Application in Expansion Microscopy
Source: ACS Omega. 2024 Aug 28;9(36):37687–701. doi: 10.1021/acsomega.4c02475 (PMC11391540; doi:10.1021/acsomega.4c02475)
Supplement: Supplementary file 1 — ao4c02475_si_001.pdf [file ao4c02475_si_001.pdf]

Stimuli-Responsive Phosphate Hydrogel: A Study on Swelling Behavior, Mechanical Properties and Application in Expansion Microscopy

Yokly Leng[1], Collin N. Britten[2], Fatema Tarannum[2], Kayla Foley[2], Christopher Billings[3], Yingtao Liu[3], and Keisha B. Walters[2]*

[1] School of Chemical, Materials, and Biological Engineering, University of Oklahoma, Norman, OK 73019
[2] Ralph E. Martin Department of Chemical Engineering, University of Arkansas, Fayetteville, AR 72701
[3] School of Aerospace and Mechanical Engineering, University of Oklahoma, Norman, OK 73019

*Corresponding Author: keishaw@uark.edu, 479-575-4834

S1. Swelling and Equilibration of Hydrogels

To prepare the phosphate buffer solution (PBS), 8.0 g of NaCl, 1.15 g of Na_2HPO_4 anhydrous, 0.2 g KCl, and 0.2 g of KH_2PO_4 were placed in a 1 L beaker, and then Type 1 ultrapure water was added to obtain a volume 800 mL. The solution was stirred using a magnetic stir plate for at least 10 min. After complete dissolution, additional Type 1 ultrapure water was added to obtain 1 L of PBS solution. A *pH* meter measured the resultant *pH* value of 7.4.

After polymerization, the hydrogels were placed on pre-weighed metal mesh weigh boats to obtain their initial masses before swelling. The boats were then submerged in Dulbecco's *pH* 7.4 PBS for 12 h to neutralize the phosphate groups. Sample weigh boats containing these swollen hydrogels were then carefully moved to a new container containing Type I ultrapure water for 12 h. Swelling experiments were carried out under various conditions as reported in the manuscript, to assess the swelling and equilibration behavior of the hydrogels .

S2. Preparation of Salt Solutions

Salt solutions were prepared to use in evaluating salt-induced swelling in the PMOEP hydrogels. NaCl, KCl, $MgCl_2$, and $CaCl_2$ salt solutions were prepared by dissolving the respective solid salts in Type 1 ultrapure water to achieve 0.1 M, 0.5 M, and 1 M solution concentrations. For example, to make a 0.1 M NaCl solution, 1.1688 g of NaCl pellets were added to a beaker. Type 1 ultrapure water was added to reach 200 mL and the mixture stirred for 30 min using a magnetic stir plate. Similarly, KCl, $CaCl_2$, and $MgCl_2$ were separately dissolved into water slowly to avoid overheating due to their exothermic dissociation in water. The hydrogels on the tared boats were then placed on a petri dish, and the salt solutions added. Masses of the hydrogel samples were measured at specified time intervals to determine the rate of swelling. After 12.5 h, the salt solutions were replaced with fresh solutions at the same initial concentration. The small jump noted at 12.5 h in the rate of swelling plot (Figs. S1 and S2) is the result of the salt solution replacement, as the solution concentration has changed due to uptake by the hydrogel over the prior 12.5 h.

S3. Induced Swelling Procedure of Hydrogels

PMOEP hydrogels were initially swollen in *pH* 7.4 PBS for 12 h followed by Type 1 ultrapure water for 12 h, with the solutions being replaced by fresh solvent every 4 h. Next, the solvent was replaced by 0.1 M NaCl, KCl, $CaCl_2$, or $MgCl_2$ solutions. The mass change study was conducted for 48 h, replacing the salt solutions every 9 h. The mass of the hydrogel was measured at discrete time intervals until the mass of the samples and therefore swelling had equilibrated.

S4. *pH* Adjustments of PBS

The *pH* values of different immersion solutions were adjusted to investigate the impact of pH on the swelling behavior of hydrogels, in addition to ionic strength and valency. To lower the *pH*, HCl was added to the *pH* 7.4 PBS until *pH* values of 1.5, 4, and 6.5 were obtained. To increase the *pH*, 10 M NaOH solution was made by dissolving 40 g of NaOH in 100 mL of Type 1 ultrapure water. The 10 M NaOH was then slowly added with mixing to *pH* 7.4 PBS until a *pH* of 10 was measured. The Type 1 ultrapure water was obtained from a Millipore Synergy UV Water Purification System delivering a 5.662 *pH* value and conductivity of 0.055 µS/cm. Each weighed/tared wire boat and hydrogel sample were placed in a petri dish along with the pH-adjusted solutions. The hydrogel masses were measured accordingly.

S5. Temperature Adjustment for Different Solutions

0.1 M NaCl, 1 M $CaCl_2$, Type 1 ultrapure water, and PBS solutions at *pH* values of 1.5, 6.5, and 10 were selected for further investigations of the hydrogels at elevated temperatures of 30 °C and 40 °C. Solutions were placed in an oven for at least 30 min to reach the target temperature before addition to the petri plates holding the weighed/tared wire boat and hydrogel. These petri dishes were covered and placed in the oven for this 24 h study. The hydrogels masses were measured at specified time intervals.

S6. *pH* Adjustments for MOEP Solutions with Different Volume Compositions

The aqueous monomer solutions (50%/50% v/v MOEP/H_2O) have very low *pH*, ca. 0.7, due to the contribution of phosphoric acid present in commercial MOEP. NaOH was used to increase the *pH* of MOEP solutions up to values of 1 and 1.5. It was observed that the MOEP solution *pH* dropped with an addition of a small amount of NaOH, contradicting what was expected when a strong base is added to an acidic solution. This seemingly unexpected *pH* change can be explained by hydrolysis, which results in the formation of additional hydrogen ions and a resultant reduction in *pH*. Regardless of concentration, adding a small amount of NaOH into MOEP solution caused a decline in *pH*. Native *pH* values of 40%/60% v/v and 60%/40% v/v MOEP/H_2O solutions were 1.04 and 0.4, respectively. It was observed that the quantity of NaOH required to attain *pH* values of 1 and 1.5 for 50%/50% v/v and 60%/40% v/v MOEP/H_2O solutions was relatively similar.

S7. Preparation of Coverslips and Use for Cell Seeding in PMOEP Polymerization

Coverslips were first cleaned with piranha solution for a minimum of 5 min before being rinsed at least 3X and soaked in Type 1 ultrapure water to remove excess piranha solution before seeding the cells. Gelatin solutions (2 mg/mL) were prepared and treated with UV light for 10 min along with 12-well plates. The prepared coverslips were then added into the wells of the 12-well plate, 1 mL of gelatin solution was added to each well, and then topped with a coverslip for 2 h. This procedure was used to seed 4T1 mouse breast cancer epithelial cells onto coverslips to the

desired density; the cells were allowed to grow for 24 to 48 h. Prior to using the cell seeded coverslip in the aqueous ARGET-ATRP polymerizations of PMOEP, a fixation solution consisting of 3% v/v paraformaldehyde (PFA) and 0.1% v/v glutaraldehyde in phosphate buffer solution was used to kill the cells for 15 minutes. The dead cells were then washed with phosphate buffer solution (PBS) three times. The cells were then quenched through treatment with a 1 mg/mL solution of sodium borohydride in PBS for 10 minutes followed by the addition of 1 mL of 100 mM glycine solution in PBS for 20 minutes. The quenching solution was removed by washing with PBS 3X. The cells were finally treated with a post-fixation solution consisting of 300 μL of MOEP and 440 μL of 16% PFA in 1 mL of PBS and 8.26 mL of water. Aqueous ARGET ATRP was then performed in accordance with the procedure discussed in the main document (Section 2.3). Schematic illustration of the cell seeding procedure is presented in main manuscript (Scheme 2a).

S8. Cell Digestion of Hydrogels

After the polymerization, the hydrogels were prepared for cell digestion. The glass slides covering the wells were removed, and the coverslips were transferred into a well plate. A digestion buffer containing 8 U/mL proteinase K, 1 mM EDTA, 50 mM Tris-Cl, 46.7 mg/mL NaCl, and 50 mg/mL Triton X-100 was then added to the well and placed on a 55 °C hot plate for 6 h to digest. The hydrogel was then removed from the coverslip and allowed to swell in PBS and Type 1 ultrapure water. Schematic illustration of cell digestion is presented in main manuscript (Scheme 2b).

S9. Supplemental Rate of Swelling Kinetics

Additional rate of swelling plots are included to provide further visual clarity and comparison of the swelling rates for different salt species (Figure S1) and salt concentrations (Figure S2).

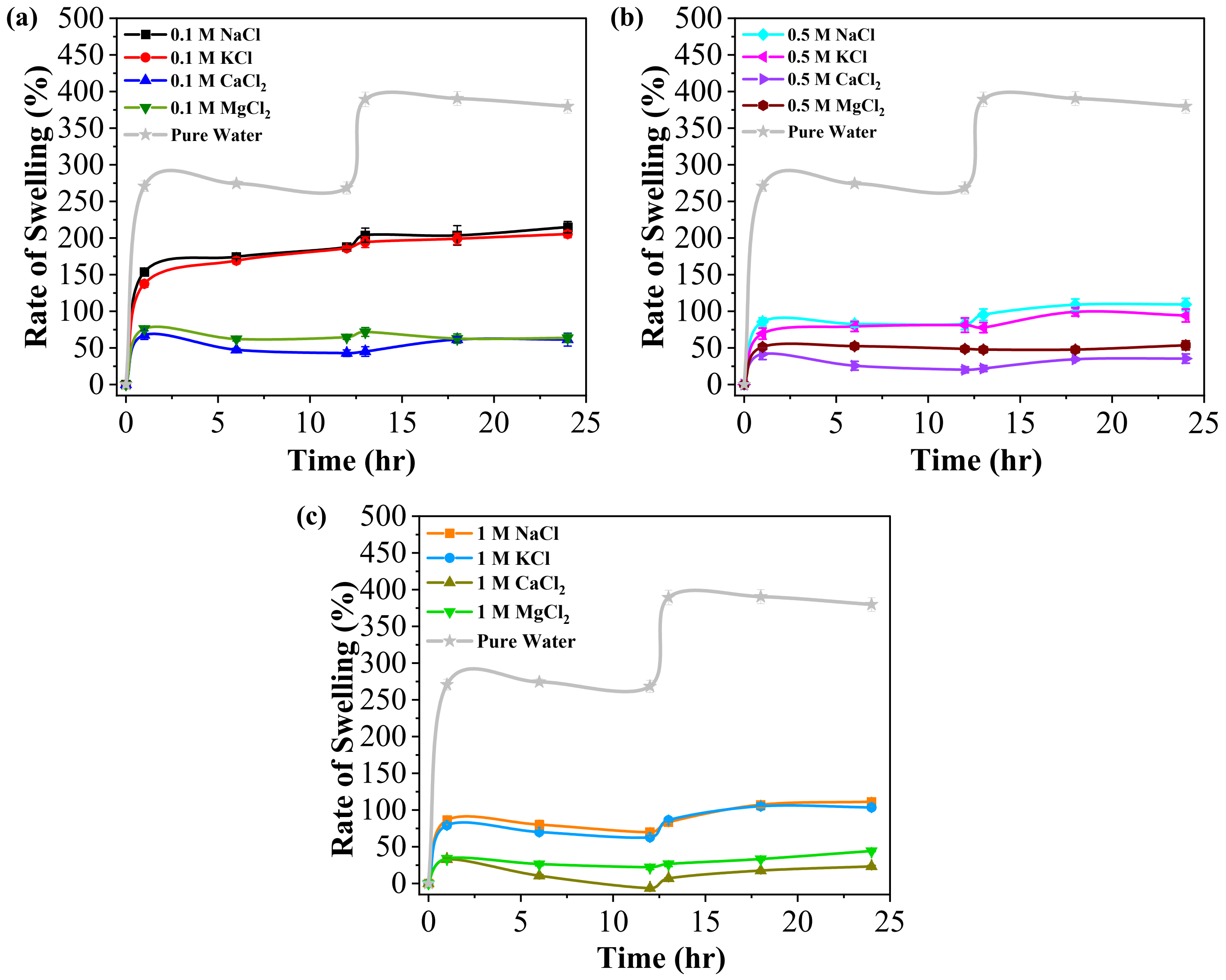

Fig. S1 Rate of swelling for PMOEP (containing 50% MOEP) in salt solutions at (a 0.1 M, (b 0.5 M, and (c 1 M concentrations.

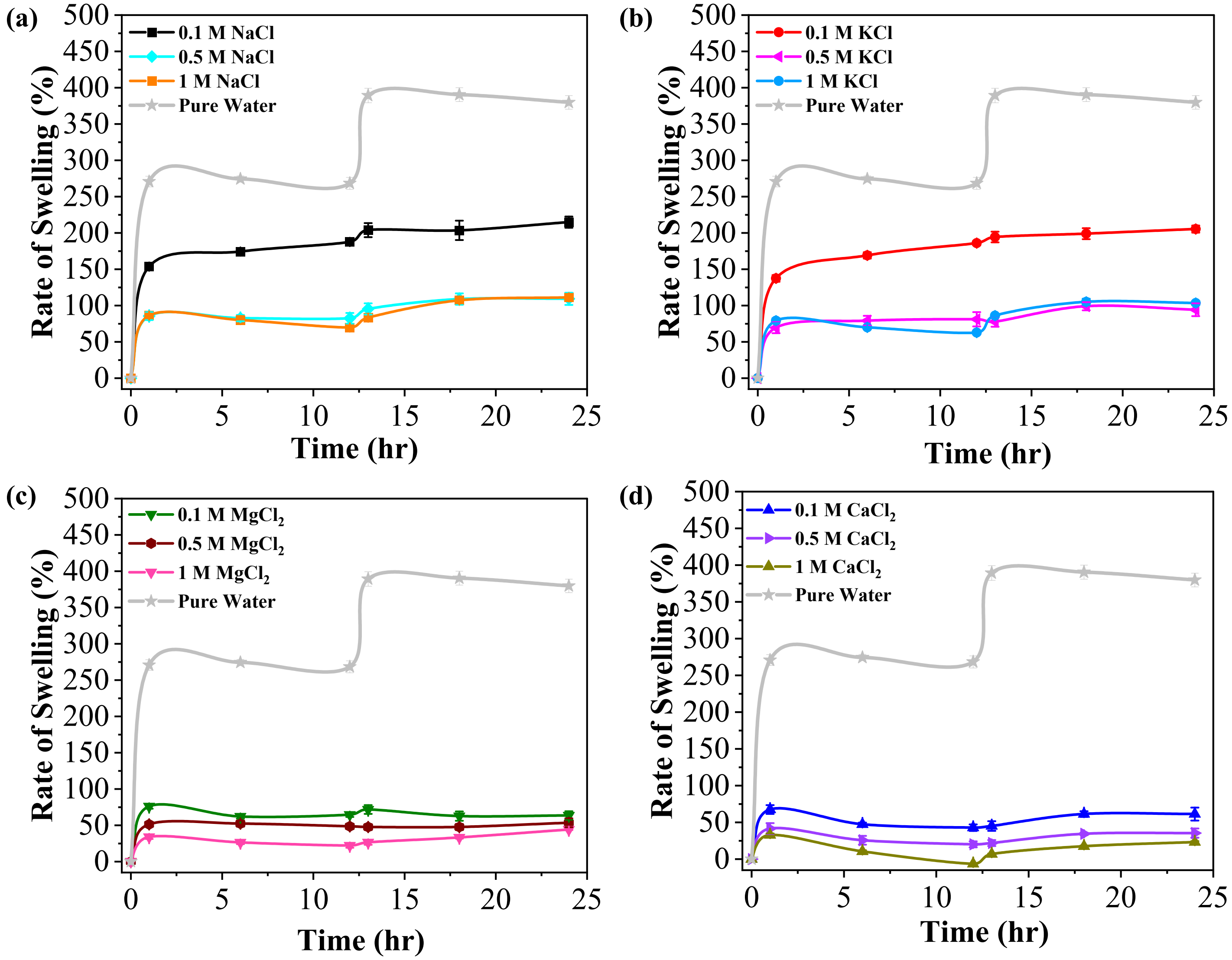

Fig. S2 PMOEP rates of swelling in different concentration solutions of (a NaCl, (b KCl, (c $MgCl_2$, and (d $CaCl_2$.

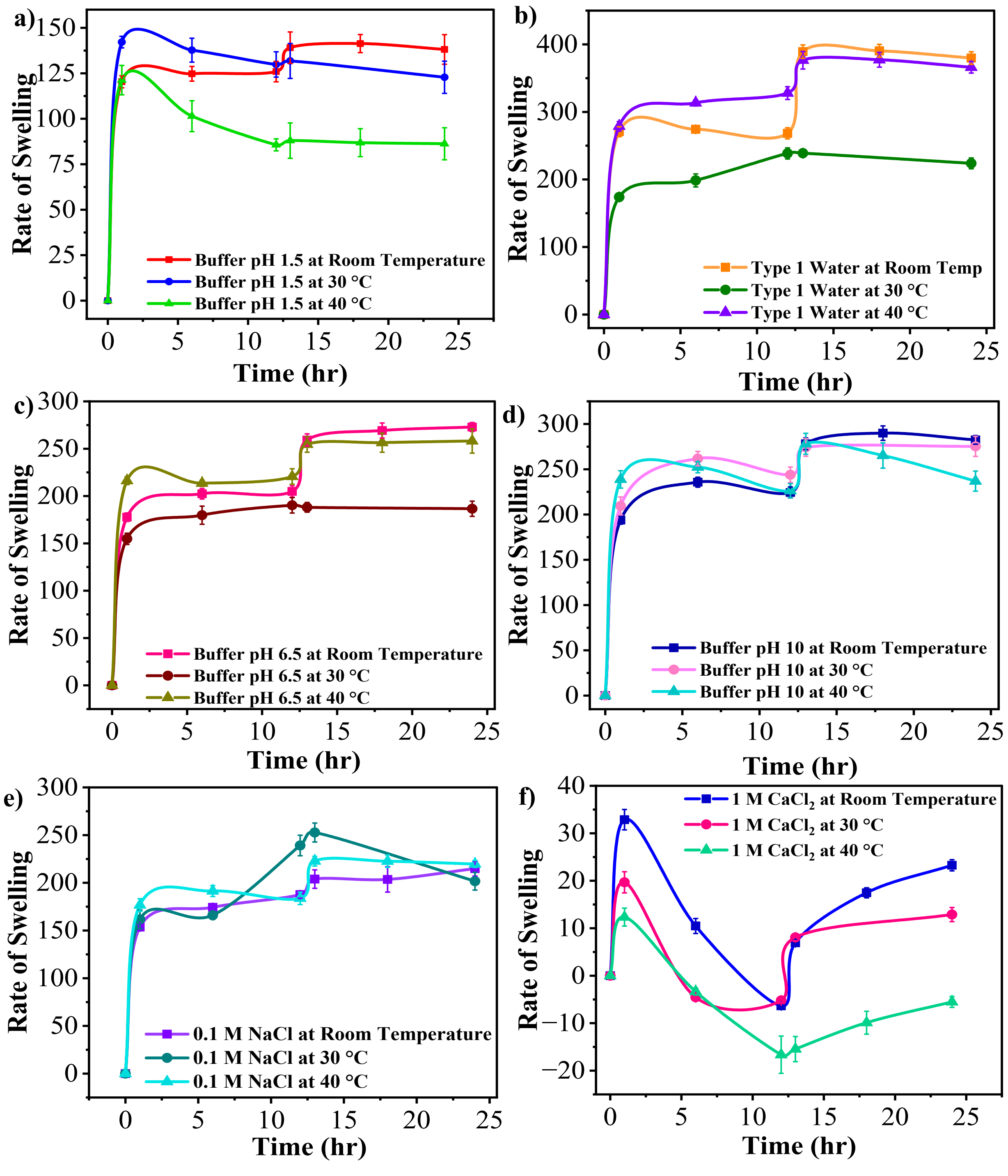

Fig. S3 Swelling rates as a function of time for PMEOP (containing 50% MOEP) in (a) *pH* 1.5 buffer, (b) Type 1 ultrapure water, (c) *pH* 6.5 buffer, (d) *pH* 10 buffer, (e) 0.1 M NaCl, and (f) 1.0 M $CaCl_2$ at RT, 30 °C, and 40 °C.

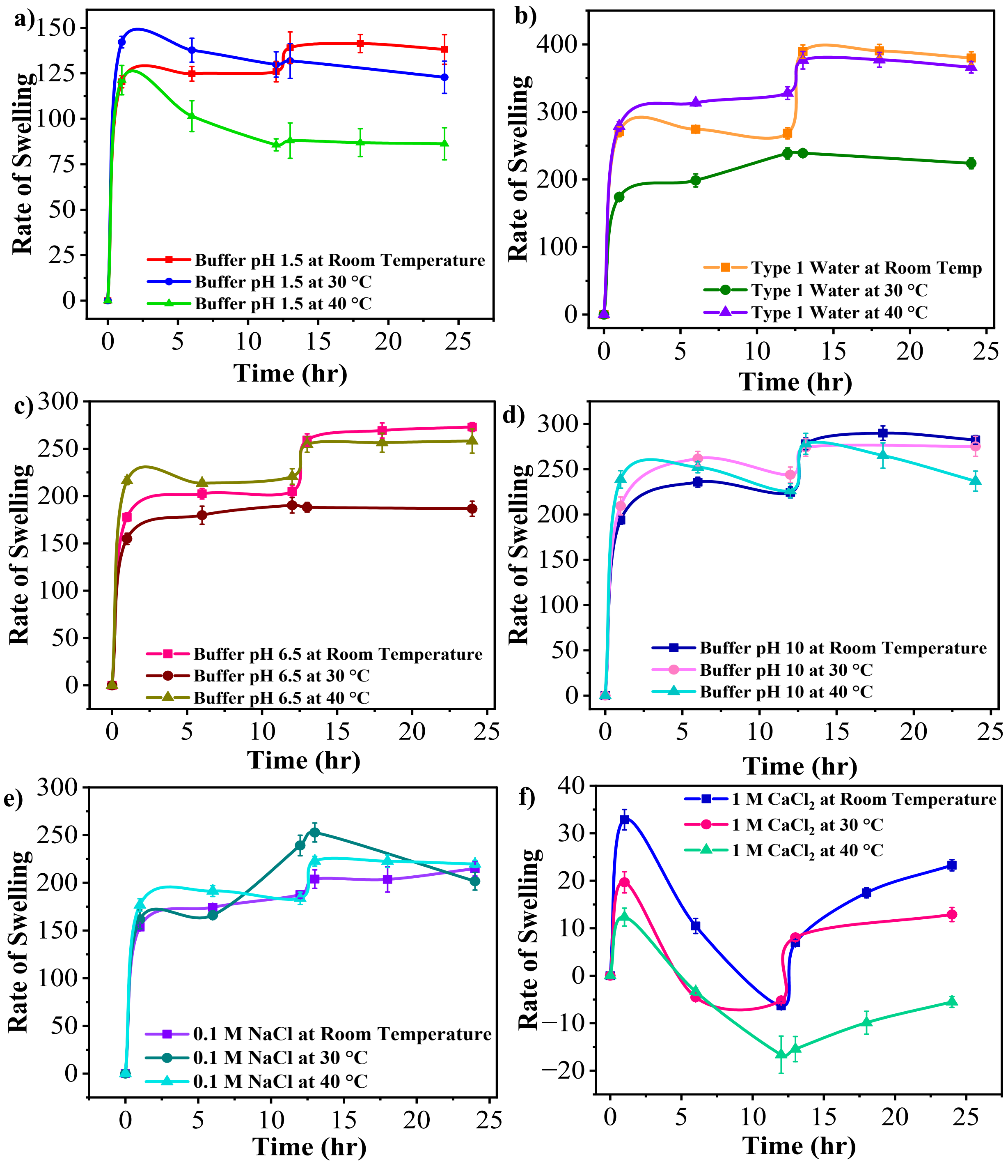

Fig. S4 Swelling rates as a function of time for PMEOP (containing 50% MOEP) in (a) *pH* 1.5 buffer, (b) Type 1 ultrapure water, (c) *pH* 6.5 buffer, (d) *pH* 10 buffer, (e) 0.1 M NaCl, and (f) 1.0 M $CaCl_2$ at RT, 30 °C, and 40 °C.

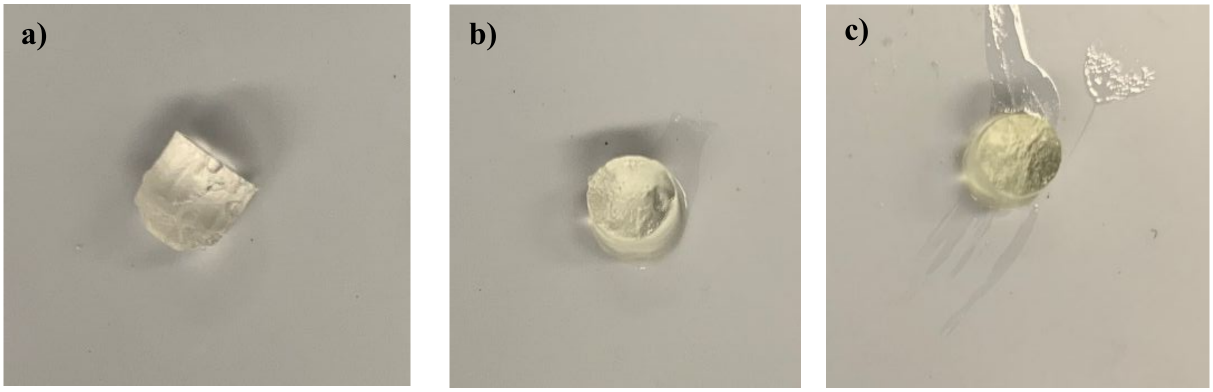

Fig. S5 Images showing the coloring of PMOEP after polymerization at 50 °C with a) native 0.7 *pH*, b) *pH* 1.0, and c) *pH* 1.5.

S10. Supplemental SEM/EDS Analyses of Cations Present on Dried Hydrogels

SEM and EDS analyses were used to confirm the presence of salts on the hydrogel surface after swelling in different salt solutions. Figure S6 shows the atomic and weight percentages of each divalent (Ca^{2+} and Mg^{2+}) and monovalent (Na^{+} and K^{+}) cation observed on the surfaces of the PMOEP hydrogels after swelling in different salt solution concentrations. Sodium (Na) surface deposition increased along with the increase in solution concentration. Magnesium (Mg) shows a similar behavior for the 0.1 M to 0.5 M increase, but did not increase with higher concentration (from 0.5 M to 1 M). Of the salts tested, potassium (K) and calcium (Ca) showed highest atomic% and weight % at 0.5 M. The drop in K and Ca contents at 1.0 M is likely attributed to equilibrium in osmotic pressure between the hydrogel surface and the solvent, resulting in a saturated hydrogel surface. This saturation prevents further binding of K and Ca ions to the hydrogel network, rendering the hydrogel unable to accept additional cations. The atomic sizes of each cation can further restrict the ability to diffuse. Na has an atomic size of 0.190 nm and shows a continuous increase in both atomic and weight percentages with increasing solution concentrations (0.1 M to 1 M) as shown in Figure S6. On the other hand, K and Ca with atomic sizes of 0.243 nm and 0.194 nm respectively, result in a decrease in their contents on the hydrogel. EDS elemental mappings of dried hydrogels after swelling in different salt concentrations are presented in supplementary information Figure S3 and Table S2-S5.

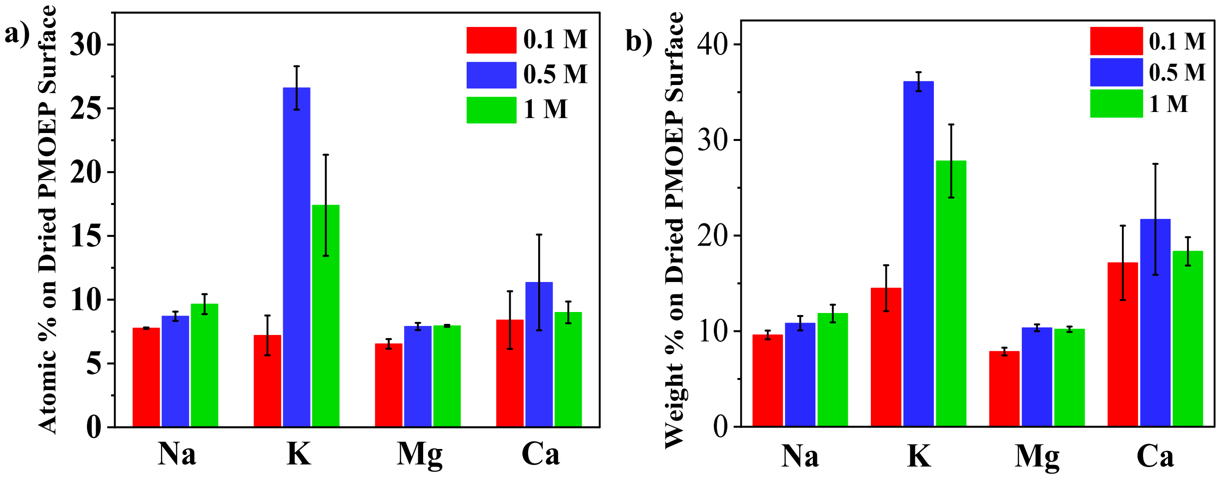

Fig. S6 a) Atomic and b) weight percentages of salt cations present on the surface of PMOEP dried hydrogel (containing 50% MOEP) from EDS data analysis.

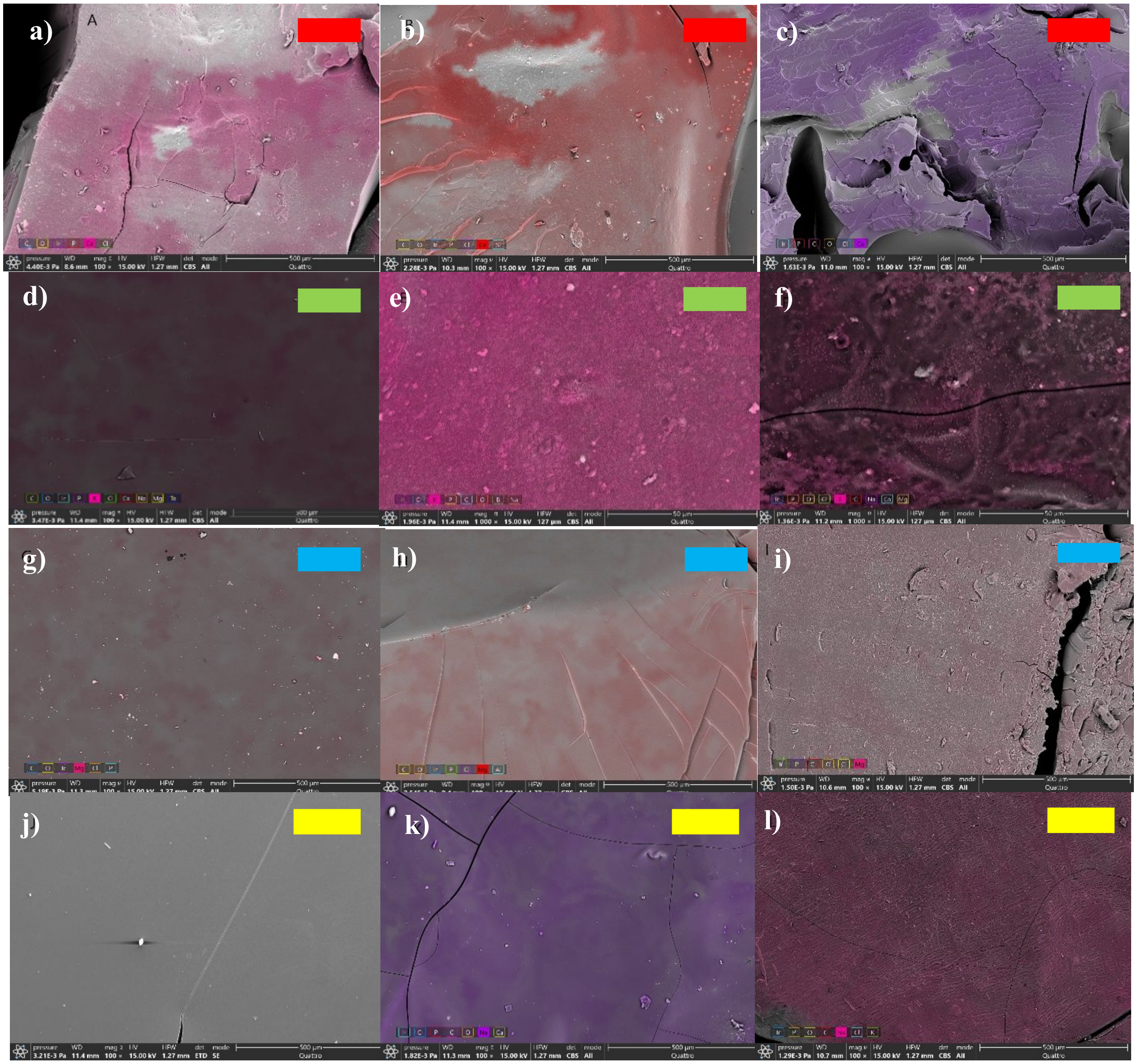

Fig. S7 SEM images showing distributions of cations on dried PMOEP hydrogel surfaces after swelling salt solutions: a) 0.1 M $CaCl_2$; b) 0.5 M $CaCl_2$; c) 1 M $CaCl_2$; d) 0.1 M KCl; e) 0.5 M KCl, f) 1.0 M KCl; g) 0.1 M $MgCl_2$; h) 0.5 M $MgCl_2$; i) 1.0 M $MgCl_2$; j) 0.1 M NaCl; k) 0.5 M NaCl; and l) 1.0 M NaCl. (Color legend: [red] $CaCl_2$; [green] KCl; [blue] $MgCl_2$; and [yellow] NaCl.)

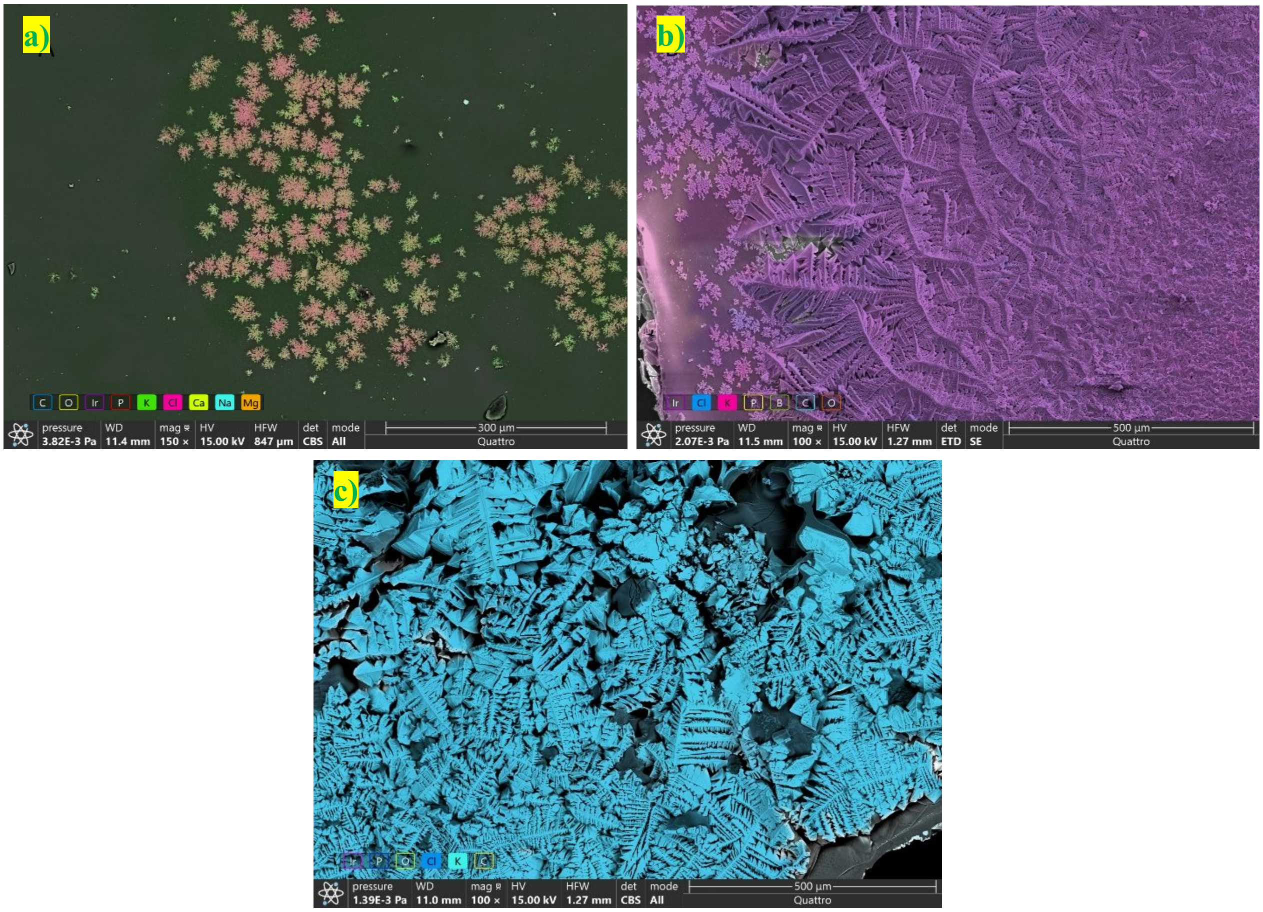

Fig. S8 ESEM images showing potassium salt precipitated on PMOEP hydrogels after drying that had been swollen in a) 0.1 M KCl, b) 0.5 M KCl, and c) 0.1 M KCl.

Table S1. Average atomic and weight percentages during deswelling of PMOEP in 0.1 M $CaCl_2$.

Element	Atomic %	Weight %
C	29.9 ± 0.2	18.63 ± 0.1
O	45.8 ± 0.2	38.1 ± 0.2
Na	0.2 ± 0.0	0.3 ± 0.0
P	15.3 ± 0.1	24.6 ± 0.1
Cl	0.3 ± 0.0	0.5 ± 0.0
Ca	8.6 ± 0.0	17.9 ± 0.1

Table S2. EDS data of dried PMOEP after swelling in $CaCl_2$ solutions.

Element	0.1M $CaCl_2$		0.5 M $CaCl_2$		1 M $CaCl_2$	
	Atomic %	Weight %	Atomic %	Weight %	Atomic %	Weight %
C	27.2 ± 0.1	16.7 ± 0.1	20.8 ± 0.2	12.2 ± 0.1	20.7 ± 0.1	12.5 ± 0.1
O	47.9 ± 0.2	39.2 ± 0.2	49.5 ± 0.3	38.4 ± 0.2	54.7 ± 0.2	44.1 ± 0.2
P	16.1 ± 0.1	25.4 ± 0.2	16.4 ± 0.2	24.4 ± 0.2	12.6 ± 0.0	19.6 ± 0.1
Cl	0.5 ± 0.0	0.8 ± 0.0	1.9 ± 0.0	3.2 ± 0.0	2.4 ± 0.0	4.3 ± 0
Ca	8.4 ± 0.1	17.2 ± 0.1	11.4 ± 0.1	21.7 ± 0.1	9.6 ± 0.0	19.4 ± 0.1
Ir	0.1 ± 0.1	0.9 ± 0.3	0.1 ± 0.0	0.3 ± 0.2	0.0 ± 0.0	0.0 ± 0.0

Table S3. EDS data of dried PMOEP after swelling in KCl solutions.

Element	0.1M KCl		0.5M KCl		1M KCl	
	Atomic %	Weight %	Atomic %	Weight %	Atomic %	Weight %
C	30.1 ± 0.2	18.8 ± 0.1	18.7 ± 0.3	7.8 ± 0.1	23.5 ± 0.2	11.8 ± 0.1
O	45.1 ± 0.2	37.55 ± 0.2	21.7± 0.4	12.1 ± 0.2	30.9 ± 0.3	20.6 ± 0.2
Na	0.2 ± 0.0	0.3 ± 0.0	0.4 ± 0.0	0.3 ± 0.0	0.5 ± 0.0	0.5 ± 0.0
Mg	0.15 ± 0.0	0.2 ± 0.0	-	-	0.2 ± 0.0	0.2 ± 0.0
P	15.0 ± 0.1	24.0 ± 0.2	28.2 ± 0.5	30.4 ± 0.5	23.6 ± 0.3	30.1 ± 0.4
Cl	0.8 ± 0.0	1.5 ± 0.0	3.0 ± 0.0	3.7 ± 0	0.9 ± 0.0	1.4 ± 0.0
K	7.2 ± 0.0	14.5 ± 0.1	-	-	17.4 ± 0.1	27.8 ± 0.2
Ca	1.5± 0.0	3.2 ± 0.1	26.6 ± 0.1	36.1 ± 0.2	2.9 ± 0.1	4.7 ± 0.1
Ir	0.0 ± 0.0	0.2 ± 0.2	1.5 ± 0.2	9.7 ± 1.6	0.5 ± 0.2	3.3 ± 1.1

Table S4. EDS data of dried PMOEP after swelling in $MgCl_2$ solutions.

Element	0.1 M $MgCl_2$		0.5 M $MgCl_2$		1 M $MgCl_2$	
	**Atomic %**	**Weight %**	**Atomic %**	**Weight %**	**Atomic %**	**Weight %**
C	26.8 ± 0.3	15.9 ± 0.1	20.3 ± 0.1	13.2 ± 0.1	21.8 ± 0.1	13.8 ± 0.1
O	36.6 ± 0.2	29.1 ± 0.2	55.0 ± 0.2	47.5 ± 0.1	50.2 ± 0.2	42.4 ± 0.1
Mg	6.5 ± 0.0	7.9 ± 0.0	7.9 ± 0.0	10.4 ± 0.0	8.0 ± 0.0	10.2 ± 0
P	25.6 ± 0.1	39.4 ± 0.2	13.3 ± 0.1	22.2 ± 0.1	15.6 ± 0.1	25.4 ± 0.1
Cl	4.4 ± 0.0	7.7 ± 0.0	3.6 ± 0.0	6.9 ± 0.0	4.5 ± 0.0	8.3 ± 0.0

Table S5. EDS data of dried PMOEP after swelling in NaCl solutions.

Element	0.1 M NaCl		0.5 M NaCl		1 M NaCl	
	**Atomic %**	**Weight %**	**Atomic %**	**Weight %**	**Atomic %**	**Weight %**
C	30.0 ± 0.2	19.4 ± 0.1	30.1± 0.2	19.6 ± 0.1	30.1 ± 0.2	19.3 ± 0.2
O	40.5 ± 0.2	34.9 ± 0.1	41.3± 0.2	35.9 ± 0.2	38.8 ± 0.2	33.1± 0.2
Na	7.8 ± 0.0	9.6 ± 0.0	8.7± 0.0	10.8 ± 0.0	9.7 ± 0	11.9 ± 0.0
P	20.8 ± 0.1	34.4 ± 0.1	19.1 ± 0.1	32.0 ± 0.1	20.1 ± 0.1	33.2 ± 0.1
Cl	0.9 ± 0.0	1.7 ± 0.0	0.7 ± 0.0	1.5 ± 0.0	1.2 ± 0	2.2 ± 0
Ca	-	-	0.1 ± 0.0	0.3 ± 0.0	-	-
K	-	-	-	-	0.3 ± 0	0.6 ± 0.0

S11. Microscopy Images of Hydrogel Samples Showing Live and Dead rBMSC

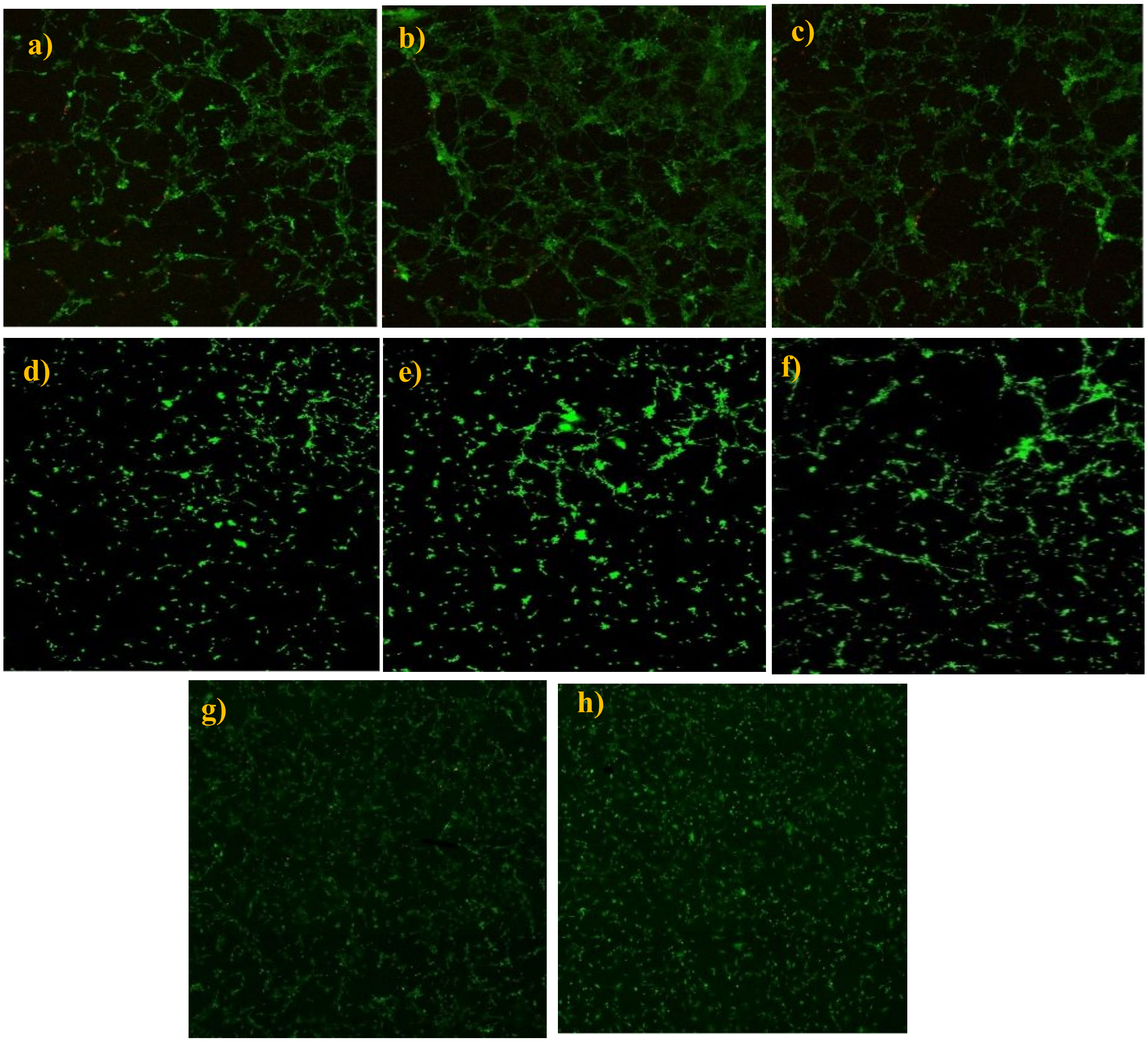

Fig. S9 Microscopy images showing live and dead rBMSCs after exposure to hydrogels polymerized at various conditions: a) 30 °C *pH* 0.5; b) 30 °C *pH* 1.0; c) 30 °C *pH* 1.5; d) 40 °C *pH* 0.5; e) 40 °C *pH* 1.0; f) 40 °C *pH* 1.5; g) 50 °C *pH* 0.5; and h) 50 °C *pH* 1.0.

S12. DSC Analysis of PMOEP

A TA Instruments Q1000 Differential Scanning Calorimeter (DSC) was used to measure the heat flow from two different samples as a function of temperature. A small sample of swollen PMOEP hydrogel was cut off using a sharp blade and placed in a weighed DSC cell. The DSC cell with the sample was then weighed and placed in the DSC chamber for thermal analysis. A second sample was similarly prepared except for the addition of Type 1 ultrapure water into the DSC cell before sealing and measuring the sample + pan mass. Each sample was subjected to heating and cooling cycles at a 10 °C/min temperature gradient between 40 - 150 °C.

DSC was used to observe the thermal behavior of PMOEP hydrogel. Figure S10 shows an endothermic peak at 113.89 °C for the sample prepared without water and 122 °C for the sample with Type 1 ultrapure water added. These peaks correspond to the loss of water from the hydrogel network as water evaporates around 100 °C [1]. DSC shows that the hydrogel exhibited greater stability in water compared to its dry state, as at higher temperature of the interaction of water and the hydrogel is observed. For the sample with water added, the exotherm is broader with the hydrogel starting to absorb energy at 102.10 °C which continues until 123 °C.

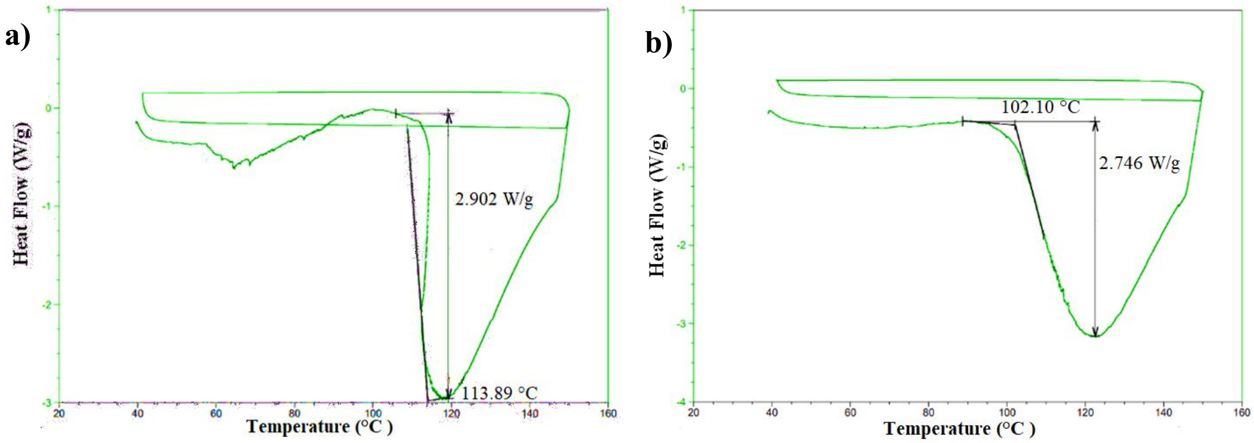

Fig. S10 DSC analysis for PMOEP samples a) prepared without additional water and b) with water added.

REFERENCES

(1) Shivakumara, L. R.; Demappa, T. Synthesis and swelling behavior of sodium alginate/poly (vinyl alcohol) hydrogels. *Turkish Journal of Pharmaceutical Sciences* **2019**, *16* (3), 252.
